# *In silico* functional analyses and discovery of survival-associated microRNA signatures in pediatric osteosarcoma

**DOI:** 10.18632/oncoscience.85

**Published:** 2014-09-23

**Authors:** Patricia C. Sanchez-Diaz, Tzu-Hung Hsiao, Yi Zou, Aaron J. Sugalski, Josefine Heim-Hall, Yidong Chen, Anne-Marie Langevin, Jaclyn Y. Hung

**Affiliations:** ^1^ Greehey Children’s Cancer Research Institute, University of Texas Health Science Center at San Antonio, San Antonio, Texas, USA; ^2^ Division of Hematology and Oncology, Department of Pediatrics, University of Texas Health Science Center at San Antonio, San Antonio, Texas, USA; ^3^ Department of Pathology, University of Texas Health Science Center at San Antonio, San Antonio, Texas, USA; ^4^ Cancer Therapy and Research Center, University of Texas Health Science Center at San Antonio, San Antonio, Texas, USA; ^5^ Department of Epidemiology and Biostatistics, University of Texas Health Science Center at San Antonio, San Antonio, Texas, USA; ^6^ Current address: Rosenberg School of Optometry, University of the Incarnate Word, San Antonio, Texas, USA

**Keywords:** osteosarcoma, microRNA expression, prognosis, pathways, pediatric cancers

## Abstract

**Purpose:**

Osteosarcoma is the most common bone tumor in children, adolescents, and young adults. In contrast to other childhood malignancies, no biomarkers have been consistently identified as predictors of outcome. This study was conducted to assess the microRNAs(miRs) expression signatures in pre-treatment osteosarcoma specimens and correlate with outcome to identify biomarkers for disease relapse.

**Results:**

A 42-miRs signature whose expression levels were associated with overall and relapse-free survival waas identified. There were 8 common miRs between the two sets of survival-associated miRs. Bioinformatic analyses of these survival-associated miRs suggested that they might regulate genes involved in ubiquitin proteasome system, TGFb, IGF, PTEN/AKT/mTOR, MAPK, PDGFR/RAF/MEK/ERK, and ErbB/HER pathways.

**Methods:**

The cohort consisted of 27 patients of 70% Mexican-American ethnicity. High-throughput RT-qPCR approach was used to generate quantitative expression of 754 miRs in the human genome. We examined tumor recurrence status, survival time and their association with miR expression levels by Cox proportional hazard regression analysis. TargetScan was used to predict miR/genes interactions, and functional analyses using KEGG, BioCarta, Gene Ontology were applied to these potential targets to predict deregulated pathways.

**Conclusions:**

Our findings suggested that these miRs might be potentially useful as prognostic biomarkers and therapeutic targets in pediatric osteosarcoma.

## INTRODUCTION

Osteosarcoma is the most common bone malignancy affecting approximately 400 children, adolescents, and young adults in the United States each year, and for which no appreciable improvement in outcome has been attained in the past 20 years, indicating that we have reached the limits of classical non-targeted therapies. Despite extensive mutilating surgery followed by aggressive chemotherapy, the survival rate for this malignant bone cancer is just 60-70%. Moreover, 40% of patients with osteosarcoma experience metastatic recurrent disease, in which case the survival rates are even lower. For the survivors, most develop debilitating long-term side effects after intensive curative treatment that impact their quality of life. Osteosarcoma arises primarily in the metaphysis of the long bones with a predilection for the areas of most intense bone growth in skeletally immature patients, i.e. distal femur, proximal tibia and proximal humerus. The peak incidence is seen in the second decade of life and seems to correlate with the growth spurt associated with pubertal development [1]. A monograph on cancer in the adolescent and young adult (AYA) population published by the NCI in 2006 revealed that Hispanics have the highest incidence of osteosarcoma in the 15 to 29-age group [2]. A retrospective analysis of the characteristics and outcome in a cohort of Hispanics children and AYA patients of predominately Mexican-American ethnicity with localized high-grade osteosarcoma of the extremity finds a striking increased rate of relapse in the preadolescent patients (before the age of 12). The study also finds that percentage of tumor necrosis after neoadjuvant chemotherapy is not directly predictive of outcome for this population of homogenously Mexican-American ancestry [3].

In contrast to other childhood malignancies, very few clinically useful indicators have been consistently identified as putative predictors of outcome. To this date, incomplete surgery remains the most important negative prognostic indicator, followed by poor response to preoperative chemotherapy, primary metastases, and axial location [4-8]. While osteosarcoma has wide-ranging histologic appearances, proliferation of malignant mesenchymal cells and production of osteoid and/or bone by the tumor cells are key features and are mandatory for clinical diagnosis. The molecular events underpinning the biology of osteosarcoma remain poorly understood but there is evidence that osteosarcoma is a differentiation disease where normal osteoblast differentiation from the mesenchymal stem cell is disrupted as a result of genetic and epigenetic changes [9-11]. In addition, numerous structural and numerical cytogenetic abnormalities have been described [12]. Yet none have been consistently predictive of survival or response to chemotherapy.

It is well known that microRNA (miR) deregulation is often associated with many cancers [13]. miRs signatures have been suggested to be a better predictor of outcome that mRNA expression profiles. A comparison between miR profiling and mRNA profiling shows that a molecular signature consisting of about 200 miRs is more accurate in classifying highly malignant, poorly differentiated tumors than mRNA profiles containing more than 20,000 genes [14]. *In silico* analyses indicate that a single miR likely regulates over 100 genes [15].

In this study, we assessed the miR expression profiles in 27 patients diagnosed with osteosarcoma localized to the extremities. The miR signatures of the pre-treatment tumor specimens were used to correlate with clinical outcome. Using Cox proportional hazard regression analyses, 32 miRs were identified to be associated with overall survival (OV-miRs), and 18 miRs were shown to predict relapse (RE-miRs). We then applied bioinformatics methods to predict pathways and functions of these survival-associated miR signatures. Our findings indicated that the survival-associated miR signatures and the putative pathways they regulate might have the potential to improve clinical management and future treatment decision-making for patients with this cancer.

## RESULTS

### Clinical characteristic of the cohort

The cohort consisted of 27 patients diagnosed with osteosarcoma of the extremities. All except one patient had their tumor specimens collected and banked at the time of diagnosis (Table S1).

The Kaplan–Meier plots of gender and race are included as supplementary data (Figure S1). Although the survival curves of gender were separated, the *P* values of the log-rank test for overall and relapse-free survival did not exhibit statistical significance. Neither the survival curve nor log-rank test showed racial difference.

### Identification of survival-associated miRs

**Figure 1:**
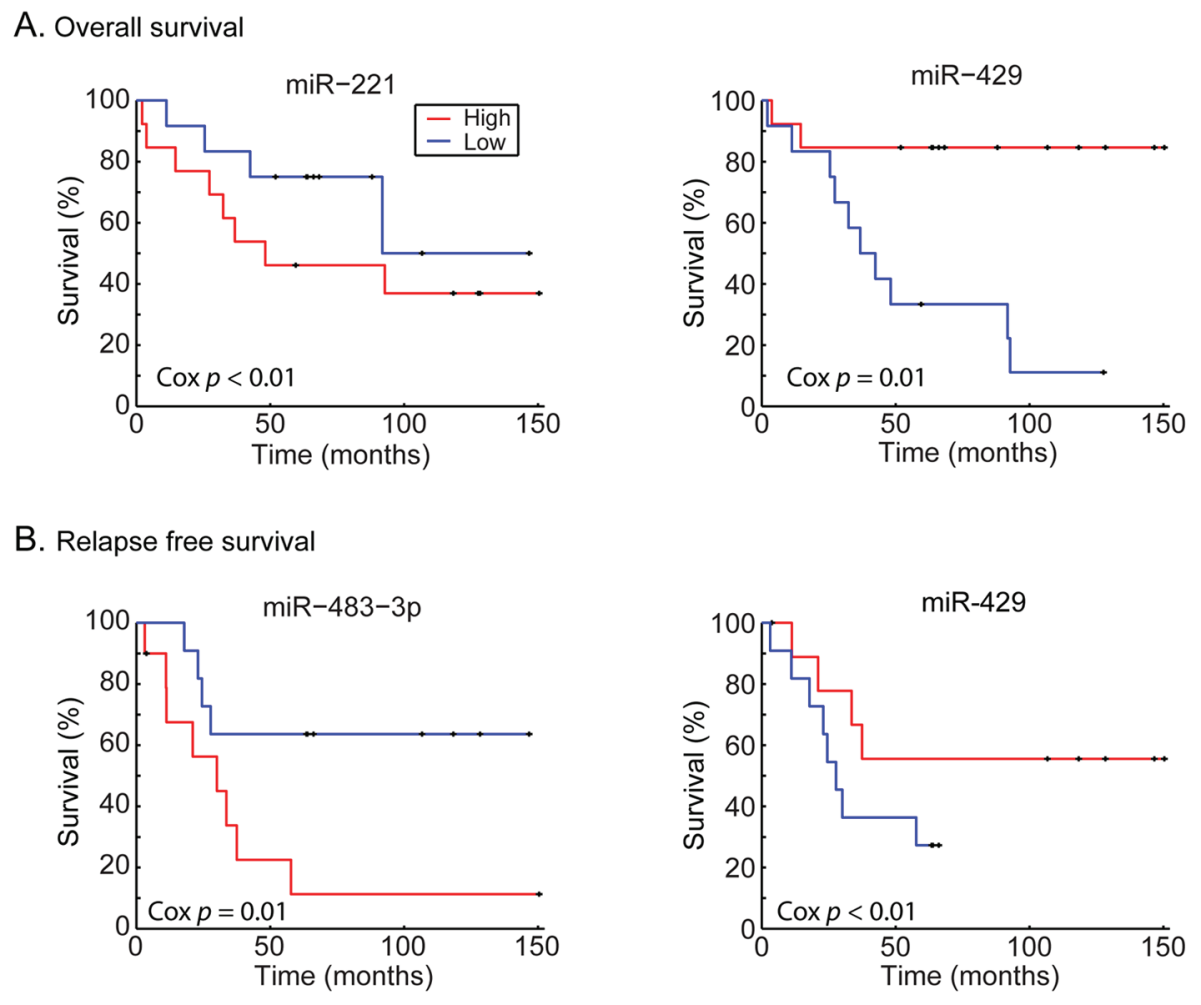
Kaplan–Meier plots of the survival-associated miRs The Kaplan–Meier plots of 4 miRs associated with patients’ survival separated by lower or higher expression abundance. The *P* values of the 4 miRs in Cox regression model were all statistically significant. In each miRs, sample was assigned to two groups, higher expression (red line) and lower expression (blue line), according to the ranking of the expression level by using the 50th percentile as criterion. (A) *hsa-miR-221* and *hsa-miR-429* were associated with overall survival with positive and negative regression coefficients. The patients with high expression of *hsa-miR-221* had worse survival rate but better survival in *hsa-miR-429*. (B) *hsa-miR-483-3p* had significant *P* value in Cox model of relapse-free survival. The patients with high expression had worse survival. *hsa-miR-429* was the most significance miRs with negative coefficients

Univariate Cox proportional analysis was performed to identify a miR signature that showed association with patients’ survival, we chose *P* value of Cox regression model <0.05 as criterion. A total of 32 and 18 miRs were associated with overall and relapse-free survival (Tables 1 and 2, respectively). In the OV-miRs, 25 out of 32 had positive hazard ratio (*β*>0), 7 had negative hazard ratio (*β*<0). Among them, *hsa-miR-221* and *hsa-miR-429* had the most significant *P* value in terms of positive and negative hazard ratio respectively. The Kaplan–Meier plots of *hsa-miR-221* and *hsa-miR-429*, shown as Figure 1A (left and right panels respectively), illustrate the survival difference between the high and low expressed miRs. In our analysis, we found that patients with a high expression of *hsa-miR-429* had better survival, whereas a poorer survival was observed in patients with a high expression of *hsa-miR-221*. For the 18 RE-miRs, 14 had positive hazard ratio and 4 negative hazards. The Kaplan–Meier plots of two of the RE-miRs are shown in Figure 1B. There were 8 overlapping miRs between the two sets of miRs (Figure S2). The possibility of selecting the 8 overlapping miR by chance was *P* < 1×10^−7^ (Fisher’s exact test).

**Table 1:** Overall survival-associated miR Cox regression parameters (*β*, confidence interval (CI) and *P* value) are provided in the table, as well miRNA’s chromosomal location.

miR	*β*	95% CI	*P* value	Chromosome
*hsa-miR-221*	0.53	[0.21 – 0.86]	0.001	Xp11.3
*hsa-miR-92a*	0.25	[0.08 – 0.42]	0.003	13q31.3 (1) or Xq26.2 (2)
*hsa-miR-770-5p*	0.5	[0.16 – 0.83]	0.003	14q32.2
*hsa-miR-628-3p*	0.24	[0.07 – 0.41]	0.01	15q21.3
*hsa-miR-505*	0.77	[0.23 – 1.31]	0.01	Xq27.1
*hsa-miR-541*	0.22	[0.05 – 0.39]	0.01	14q32.31
*hsa-miR-499-5p*	0.39	[0.09 – 0.69]	0.01	20q11.22
*hsa-miR-455-3p*	0.74	[0.15 – 1.32]	0.01	9q32
*hsa-miR-29a*	0.77	[0.14 – 1.40]	0.02	7q32.3
*hsa-miR-876-5p*	0.15	[0.02 – 0.28]	0.02	9p21.1
*hsa-miR-890*	0.18	[0.03 – 0.34]	0.02	Xq27.3
*hsa-let-7c*	0.64	[0.10 – 1.18]	0.02	21q21.1
*hsa-miR-545*	0.47	[0.06 – 0.88]	0.02	Xq13.2
*hsa-miR-486-3p*	0.19	[0.02 – 0.35]	0.03	8p11.21
*hsa-miR-622*	0.38	[0.04 – 0.73]	0.03	13q31.3
*hsa-miR-1255b*	0.33	[0.03 – 0.63]	0.03	4p14 (1) or 1q24.2 (2)
*hsa-miR-483-3p*	0.29	[0.03 – 0.56]	0.03	11p15.5
*hsa-miR-30c*	0.19	[0.02 – 0.37]	0.03	1p34.2 (1) or 6q13 (2)
*hsa-miR-485-3p*	0.24	[0.01 – 0.48]	0.04	14q32.31
*hsa-miR-21*	0.4	[0.01 – 0.79]	0.04	17q23.1
*hsa-miR-374b*	0.18	[0.00 – 0.36]	0.04	Xq13.2
*hsa-miR-573*	0.48	[0.01 – 0.95]	0.05	4p15.2
*hsa-miR-1282*	0.93	[0.02 – 1.83]	0.05	15q15.3
*hsa-miR-656*	0.43	[0.01 – 0.86]	0.05	14q32.31
*hsa-miR-202*	0.34	[0.00 – 0.69]	0.05	10q26.3
*hsa-miR-429*	−0.29	[−0.51 – −0.07]	0.01	1p36.33
*hsa-miR-126*	−0.36	[−0.64 – −0.08]	0.01	9q34.3
*hsa-miR-888*	−0.56	[−1.02 – −0.10]	0.02	Xq27.3
*hsa-miR-517b*	−0.22	[−0.41 – −0.03]	0.03	19q13.42
*hsa-miR-363*	−0.43	[−0.80 – −0.05]	0.03	Xq26.2
*hsa-miR-216b*	−0.26	[−0.50 – −0.03]	0.03	2p16.1
*hsa-miR-1285*	−0.27	[−0.53 – −0.02]	0.04	7q21.2 (1) or 2p13.3 (2)

**Table 2:** Relapse-free survival-associated miR

miR	*β*	95% CI	*P* value	Chromosome
*hsa-miR-483-3p*	0.36	[0.08 – 0.64]	0.01	11p15.5
*hsa-miR-500*	0.7	[0.14 – 1.26]	0.01	Xp11.23
*hsa-miR-545*	0.63	[0.11 – 1.14]	0.02	Xq13.2
*hsa-miR-221*	0.44	[0.08 – 0.81]	0.02	Xp11.3
*hsa-miR-30b*	0.45	[0.08 – 0.82]	0.02	8q24.22
*hsa-miR-541*	0.22	[0.03 – 0.41]	0.03	14q32.31
*hsa-miR-30a*	0.29	[0.03 – 0.55]	0.03	6q13
*hsa-miR-146b-5p*	0.83	[0.05 – 1.61]	0.04	10q24.32
*hsa-miR-668*	0.58	[0.03 – 1.13]	0.04	14q32.31
*hsa-miR-770-5p*	0.4	[0.01 – 0.79]	0.04	14q32.2
*hsa-miR-335*	0.42	[0.01 – 0.83]	0.04	7q32.2
*hsa-miR-328*	0.24	[0.01 – 0.47]	0.04	16q22.1
*hsa-miR-449b*	0.22	[0.00 – 0.43]	0.05	5q11.2
*hsa-miR-499-5p*	0.36	[0.00 – 0.71]	0.05	20q11.22
*hsa-miR-429*	−0.39	[−0.65 – −0.13]	0.003	1p36.33
*hsa-miR-151-3p*	−0.18	[−0.32 – −0.04]	0.01	8q24.3
*hsa-miR-888*	−0.83	[−1.48 – −0.17]	0.01	Xq27.3
*hsa-miR-511*	−0.24	[−0.47 – −0.01]	0.04	10p12.33

**Figure 2:**
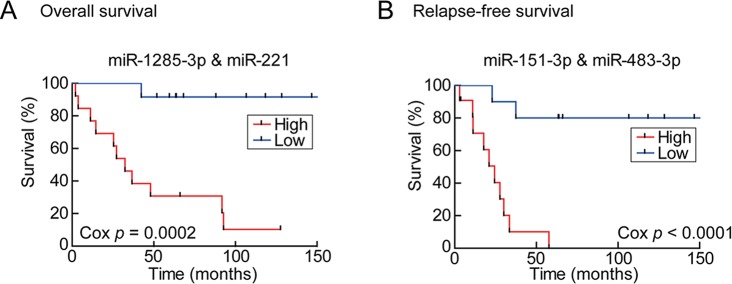
Kaplan–Meier plots of the survival-associated miR pairs The miRs pairs were identified by multi-variants Cox regression model. Each sample was assigned to two groups, higher (red line) and lower (blue line) hazard, based on the hazard values. (A) The miR pairs of *hsa-miR-1285-3p* and *hsa-miR-221* and (B) *hsa-miR-151-3p* and *hsa-miR-483-3p* (B) had statistical significance in overall and relapse-free survival.

In order to enhance the prediction power, multivariate Cox hazard regression analysis was done to assess the correlation between the survival-associated miRs pairs and patients’ survival. Using a selection criteria of *P* values < 0.01 in the miRs pair (listed in supplementary Table S2 and S3), 30 and 13 pairs of miRs were found to be associated with overall and relapse-free survival, respectively. For example, multivariate analysis improved the *P* value of the OV-miR pair *hsa-miR-221* and *hsa-miR-1285; hsa-miR-221* from 0.001 (Table 1) to 0.0003 (Table S2) and *hsa-miR-1285* from 0.04 to 0.003. The Kaplan–Meier plots of this OV-miR pair is shown on Figure 2A. In Figure 2B, RE-miR pair of *hsa-miR-483- 3p* (*P* < 0.01) and *hsa-miR-151-3p* (*P* < 0.04) showed improved predictive power (*P* < 0.0001).

### Survival-associated miR predicted target genes

**Figure 3:**
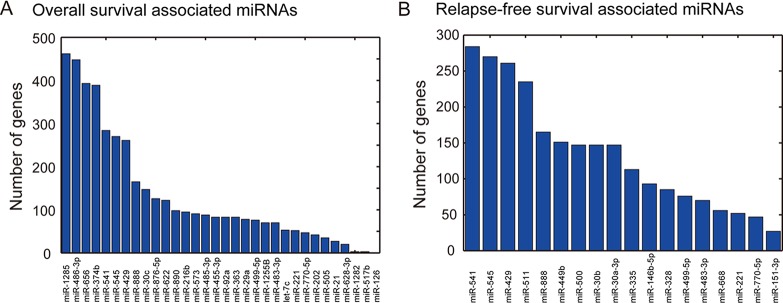
The number of genes targeted by the survival-associated miRs (A) Bar charts of the number of genes regulated by OV-miRs and (B) RE-miRs. *hsa-miR-1285* and *hsa-miR-541* had the largest number of genes (462 and 264 genes, respectively) targeted by the survival-associated miRs.

The target genes of the survival-associated miRs were predicted using TargetScan (http://www.targetscan.org). The *in silico* analysis indicated 870 and 1,059 target genes regulated by the OV-miRs and RE-miRs, respectively (supplementary Table S4A and S4B). Our analysis revealed that each OV-miR and RE-miR regulated on average 80.3 and 88.3 of the predicted genes, respectively (Figure 3). The OV-miR, *hsa-miR-1284* regulated the largest numbers of predicted genes (462). *hsa-miR-541* was the RE-miR with the most number of predicted target genes, 264.

### Enrichment analyses and pathway analysis

**Figure 4:**
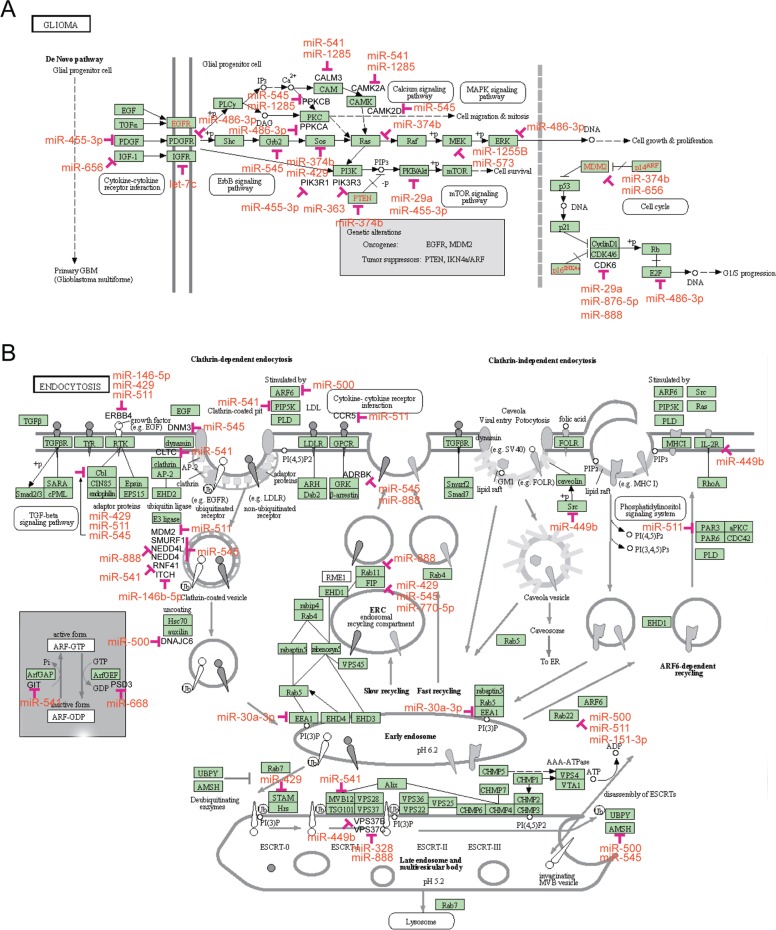
KEGG pathways enrichment analysis of the survival-associated miRs target genes (A) The GLIOMA signaling pathway of KEGG includes 26 genes that were highly regulated by the OV-miRs. Fifteen of the 42 survival-associated miRs were involved in this pathway. Eleven of the miRs (*hsa-miR-876-5p, hsa-miR-1255b, hsa-miR573, hsa-miR-29a, hsa-miR-455-3p, hsa-miR374b, hsamiR- 545, hsa-miR-486-3p, hsa-miR-541, hsa-miR-656,* and *hsa-miR-let-7c*) were of poor prognosis. miR/gene interactions included growth factors, oncogenes and tumor suppressor genes, such as *IGF-1*, *PDGF*, *CDK6*, *E2F1*, *MDM2*, RAS, SRC, AKT, ERK and *PTEN*. (B) The endocytosis pathway of KEGG was regulated by the RE-miRs. E3 ligases and deubiquitinases such as *MDM2, SMUR1, NEDD4L, NEDD4, RNF41, ITCH, CBL, UBPY,* and *AMSH* were targeted by 9 (*hsa-miR-545, hsa-miR-541, hsa-miR-511, hsa-miR-888, hsa-miR-146-5p, hsamiR- 429, hsa-miR-374b*, *hsa-miR-500,* and *hsa-miR-656*) of the predicted poor prognosis miRs.

Discovery of the function of the target genes of the survival-associated miRs and pathway enrichment analyses were performed using GO BP, KEGG and BioCarta pathways bioinformatic tools. As shown in Tables S5 and S6, the number of KEGG pathways of the OV-miRs and RE-miRs data sets was 36 and 13, respectively. In the KEGG_GLIOMA pathway, oncogenes and tumor suppressor genes, such as *KRAS*, *MDM2*, *E2F1*, *PTEN, SOS1* and *MAPK1* were regulated by the OV-miRs (Figure 4A). In the KEGG ENDOCYTOSIS, shown in Figure 4B, TGFβ signaling and the ubiquitin proteasome pathway containing genes such as ubquitin E3 ligases and *UBPY* and *AMSH* (deubiquitinating enzymes) were regulated by the RE-miRs. We identified 20 and 8 items in the BioCarta signaling pathways for OV-miRs and RE-miRs data sets, respectively (Table S7 and S8). The BIOCARTA EIF4 PATHWAY, which was the pathway regulated by eIF4e and p70 S6 Kinase and BIOCARTA CREB PATHWAY were regulated by the OV-miRs and RE-miRs, respectively (Figure S3). Several known oncogenes and tumor suppressor genes, such as p38, *ERK1*, *ERK2*, *PTEN*, and *PI3K*, were regulated by the OV-miRs in this pathway. The GO BP analysis identified 66 and 52 items with statistical significance in the OV-miRs and RE-miRs data sets, respectively (Tables S9 and S10). Our analysis showed some overlapping GO terms between the two data sets including the regulation of transcription, DNA-dependent, synaptic transmission, *in utero* embryonic development, or post-embryonic development (*P*< 10^−4^).

## DISCUSSION

Our population of children and AYA diagnosed with osteosarcoma localized to the extremities was 70% Hispanic, predominantly of Mexican American ancestry, providing us with a unique cohort. Five of these patients were preadolescent (below 12 years of age at diagnosis) of which 90% was Hispanic. Twelve patients in our cohort deceased, including the 5 preadolescents (Table S1). In our cohort, we identified 32 OV-miRs and 18 RE-miRs to be predictive of outcome. There were 8 common miRs (*hsa-miR-429*, *hsa-miR-221*, *hsa-miR- 499-5p*, *hsa-miR-888*, *hsa-miR-770-5p*, *hsa-miR-545*, *hsa-miR-541*, *hsa-miR-483-3p*) between the two sets of survival-associated miRs (Figure S2). To the best of our knowledge, there were 29 unique miRs in our 42 survival-associated miR signature that had not been reported to be associated with prognosis in osteosarcoma. This could in part be explained by the nature of our cohort, since 70% of the population treated at UTHSCSA is Hispanic of Mexican American ancestry, suggesting that the underlying tumor biology in this patients’ population may be different from previous reports. However, this possibility needs to be confirmed in a larger multiinstitutional study with patients of similar demographics. Interestingly, 5 of the 42 survival-associated miRs (*hsa-miR-770-5p*, *hsa-miR-541, hsa-miR-485-3p, hsamiR- 656, hsa-miR-668)* were encoded in the chromosome 14q32 region. These 5 miRs were associated with poor prognosis in our cohort, which is keeping with previous reports that indicate the miRs cluster at 14q32 region drives aggressiveness in several malignancies including osteosarcoma [17-23].

We also used systems biology approaches to predict functions and potential pathways regulated by the two sets of miRs. TargetScan was employed to predict the putative genes targeted by the survival-associated miRs. We applied the KEGG and BioCarta pathways and GO BP to determine *in silico* gene function and performed pathway enrichment analyses of the OV-miRs and REmiRs data sets. As well, the Fisher’s exact test was used to confirm that the potential pathways identified were not due to random chance. The computational analyses of these 42 survival-associated miRs suggested that these OV- and RE- miRs might regulate genes involved in endocytosis, the ubiquitin proteasome system (UPS), TGF beta, IGF, PTEN/AKT/mTOR, MAPK, PDGFR/Raf/MEK/ERK, and ErbB/HER pathways. Interestingly, the bioinformatics analyses of the miR signature that was predictive of poor prognosis revealed miR/gene interactions involved in biological processes such as apoptosis, migration, proliferation and cell cycle. Intriguingly, several of the predictor of poor prognosis miRs targeted genes that belong to the UPS that regulates protein homeostasis. It is well known that the UPS system includes a myriad of regulatory proteins that play essential roles in cell cycle progression, tumorigenesis, DNA transcription and repair, and differentiation [24- 26]. In addition, evidence suggests that components of this degradation machinery may act as tumor suppressors or as oncogenes in a context dependent manner [27-29]. Moreover, targeting specific components of the UPS for the modulation of protein degradation is an emerging and promising therapeutic strategy [30-32]. Strikingly, 9 of the poor prognosis associated miRs: *hsa-miR-545, hsa-miR-541, hsa-miR-511, hsa-miR-888, hsa-miR-146- 5p, hsa-miR-429, hsa-miR-374b*, *hsa-miR-500,* and *hsamiR- 656* have functions in E3 ligases and deubiquitinases (Figure 4B). Notably, 3 of these miRs (*hsa-miR-545, hsa-miR-541,* and *hsa-miR-511*) targeted more than one component of the UPS system. These observations indicated that further investigations are needed to characterize these miRs to improve our understanding of the biology of osteosarcoma and discover novel targets for therapeutic intervention.

In summary, we surveyed 27 children and AYA diagnosed with osteosarcoma localized to the extremities treated at a single institution for survival-associated miRs. Our cohort was homogenously 70% Hispanic of Mexican American ancestry. We identify a novel 42- miRs signature that was predictive of outcome. Using computational approaches we also predicted miR/genes interactions and pathways regulated by these miRs. Our findings suggested additional makers for prognostics and therapeutic intervention in these patients. Further studies are warranted to evaluate the use of these miR signatures in the clinical settings.

## MATERIALS AND METHODS

### Patient cohort

The cohort consisted of 27 children and AYA diagnosed with osteosarcoma localized to the extremities between January 1, 2000 and December 31, 2009 at the University of Texas Health Science Center at San Antonio (UTHSCSA). All patients were treated in a standardized fashion with cisplatin, doxorubicin and methotrexate with or without the addition of ifosfamide and etoposide. The study was conducted after receiving approval from UTHSCSA Institutional Review Board (IRB). Tumor tissue was obtained at the time of the initial biopsy prior to treatment. Specimens were immediately frozen and banked according to national standards in the IRB-approved UTHSCSA Oncological Musculoskeletal tumor bank. For correlation with miRs results, the data elements for demographic and socioeconomic variables such as: patient age, gender, race (NCI-standard), and ethnicity (NCI-standard), and clinical variables such as: histology subtype, location and staging of primary tumor, location and date of relapse date, and date of death were collected.

### RNA extraction and high-throughput miR expression analysis

In a −20°C cryostat, 10 slices, each 10 μm thick frozen tissue were cut (total thickness 100 μm) and placed in 50 mL centrifuge tube containing pre-chill RNA*later*-ICE solution (Ambion, Applied Biosystems). Total RNA was extracted using the mirVana miR Isolation Kit (Ambion) according to manufacturer’s instructions. Sample quantitation and integrity checks (measured as RNA Integrity Number; RIN) were performed using the Agilent 2100 Bioanalyzer (Agilent Technologies). The global profiling for miR expression was performed using the TaqMan Array Human MicroRNA Cards (Applied Biosystems), which includes Cards A (v2) and B (v3) in a 384-well format, containing a total of 754 specific assays specific to miRs in the human genome, annotated in the Sanger miRBase v14 Registry (http://microrna.sanger.ac.uk). Each card also contained 3 endogenous and 1 negative control assays. The stem-loop quantitative polymerase chain reaction (qRT-PCR) assay was performed according to the manufacturer’s protocol and described in [16]. The data collected were then processed using the Plate Utility and Automation Controller software (Applied Biosystems). For each miR, the expression level was determined by the threshold cycle method (2^−[Delta]Ct^) of relative quantification ([Delta]Ct = Ct of sample – Ct of endogenous controls of sample). Quantile normalization was applied to compensate for the technical differences present among all the samples so as to determine the real biological differences among sample.

### Data analysis

Cox hazard regression model was applied to calculate the hazard risk of the expression value of each miR. Univariate and multivariate analyses were conducted for each miR measured in the 27 patients to predict patient overall and relapse-free survival. The miRs that had *P* values < 0.05 were identified as either overall or relapsefree survival-associated miRs (OV-miRs or RE-miRs). For univariate Cox analysis, the good and poor prognosis patients were divided through 50% percentile of miR expression. Kaplan–Meier analyses were performed to visualize the predictive value of either of the individual or paired survival-associated miRs. To assess the correlation between two of the survival-associated miRs and patient survival, multivariate Cox hazard regression analysis was done. The patients were separated into two groups based on the hazard values. For patient *i* the hazard can be calculated as Equation (1)

$$HR_{i}=e^{\left(\beta_{1}x_{1,i}+\beta_{2}x_{2,i}\right)}$$

where *β_1_* and *β_2_* were the coefficients of the two miRs and *x*_1,*i*_ and *x*_2,*i*_ were the miR expression abundances of patient *i*.

### Prediction of miR targets genes and gene sets of KEGG, Biocarta pathway and Gene Ontology

miR target genes were identified based on the numbers of miR binding sites which were downloaded from TargetScan database (http://www.targetscan.org/). The gene containing more than 3 binding site of specific miR on its transcript’s sequence were considered as the miR target genes. For the gene that have multiple transcripts, the variant 1 recorded in Refseq database was selected as the presented transcript. The analyses of the gene sets of KEGG (www.genome.jp/kegg/pathway.html) and Biocarta pathways (http://www.biocarta.com/) and Biological Processes in Gene Ontology (GO BP) (http://www.geneontology.org/) performed as described in [16].

### miR regulated genes and enrichment analysis of miR regulated gene set

Pathway enrichment analysis of the survival-associated miR regulated gene set was obtained through Fisher’s exact test. The *P* value of Fisher’s exact test between the miR regulated genes and the genes in pathways can be calculated by

$$P(x>z_{l})=\sum_{h=z_{l}}^{\infty }\frac{\left(\begin{array}{c} r\\ h \end{array}\right)\left(\begin{array}{c} M-r\\ y_{l}-h \end{array}\right)}{\left(\begin{array}{c} M\\ y_{l} \end{array}\right)}$$

where *M* is the assume number of genes in the genome, *r* is the number of miR regulated genes, *y_l_* is the number of genes in gene set *l,* and *z_l_* is the number of miR regulated genes in gene set *l*. *P* value < 0.05 was considered significant.

## SUPPLEMENTARY FIGURES AND TABLES


