# Supplementary material for: In silico functional analyses and discovery of survival-associated microRNA signatures in pediatric osteosarcoma
Source: Oncoscience. 2014 Sep 23;1(9):599–608. doi: 10.18632/oncoscience.85 (PMC4278335; doi:10.18632/oncoscience.85)
Supplement: Supplementary file 1 [file oncoscience-01-0599-s001.pdf]

In silico functional analyses and discovery of survival-associated microRNA signatures in pediatric osteosarcoma

Supplementary Figures

Figure S1. The Kaplan–Meier plots of gender and race associated with A) patients' overall survival, and B) patients' relapse-free survival. The log-rank test *P* values were provided in the figures.

Figure S2. The number of OV-miRs, RE-miRs and overlapping survival-associated miRs illustrated as a Venn diagram. The possibility of selecting the 8 overlapping miR by chance is $P < 1x10^{-7}$ (Fisher's exact test).

Figure S3. miRs and their predicted BioCarta pathways. A) EIF4 pathway, and B) CREB pathway.

Supplementary Tables.

Table S1. Clinical characteristics of osteosarcoma tumors

Table S2. Overall survival-associated miR pairs

Table S3. Relapse-free survival-associated miR pairs.

Table S4. microRNA targeted genes derived from A) OV-miRs and B) RE-miRs.

Table S5. Enrichment of KEGG pathways with OV-miRs.

Table S6. Enrichment of KEGG pathways with RE-miRs

Table S7. Enrichment of BioCarta pathways with OV-miRs

Table S8. Enrichment of BioCarta pathways with RE-miRs

Table S9. Enrichment of Biological Processes in Gene Ontology with OV-miRs

Table S10. Enrichment of Biological Processes in Gene Ontology with RE-miRs

A Overall survival

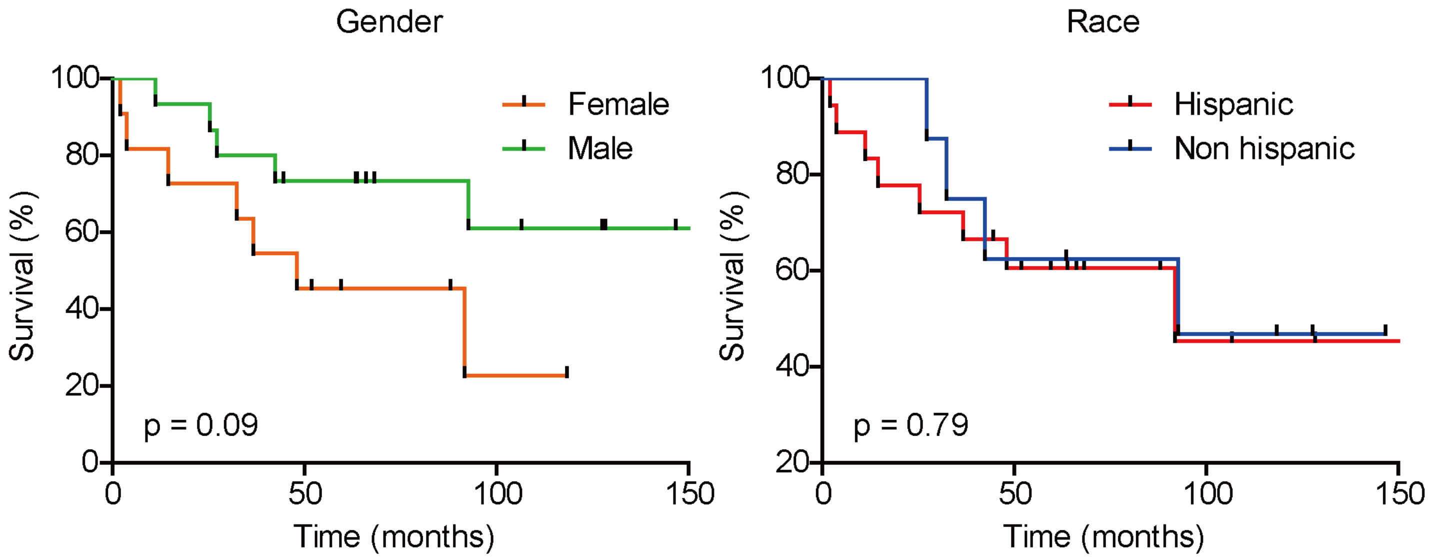

B Relapse-free survival

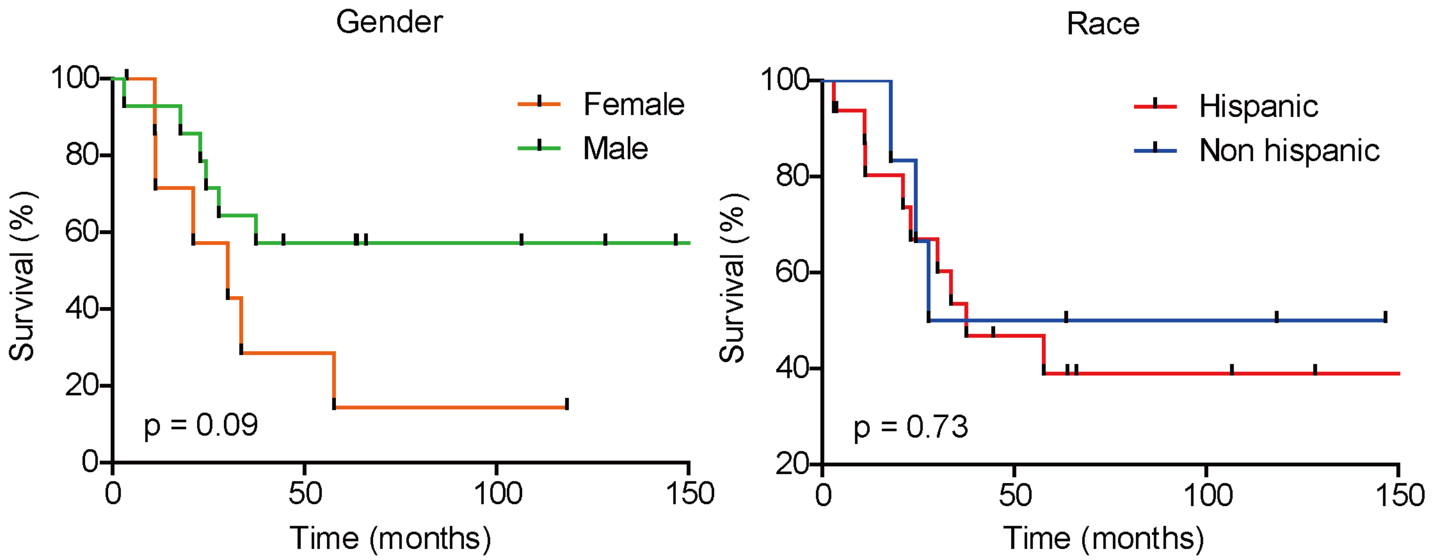

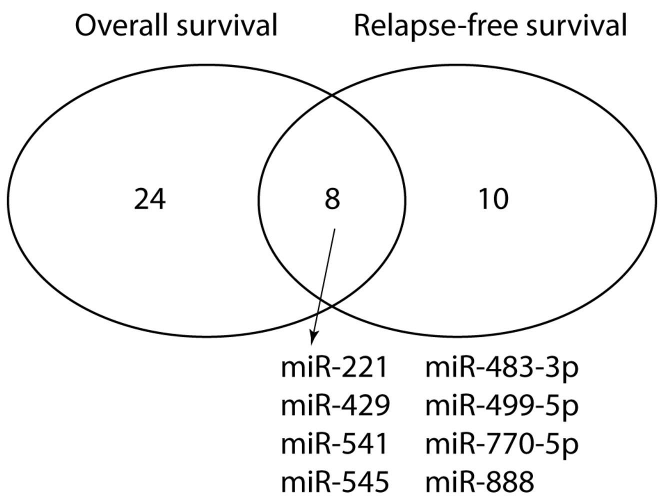
Overall survival
Relapse-free survival
24
8
10
miR-221
miR-429
miR-541
miR-545
miR-483-3p
miR-499-5p
miR-770-5p
miR-888

A. Overall survival miRNA regulated pathway

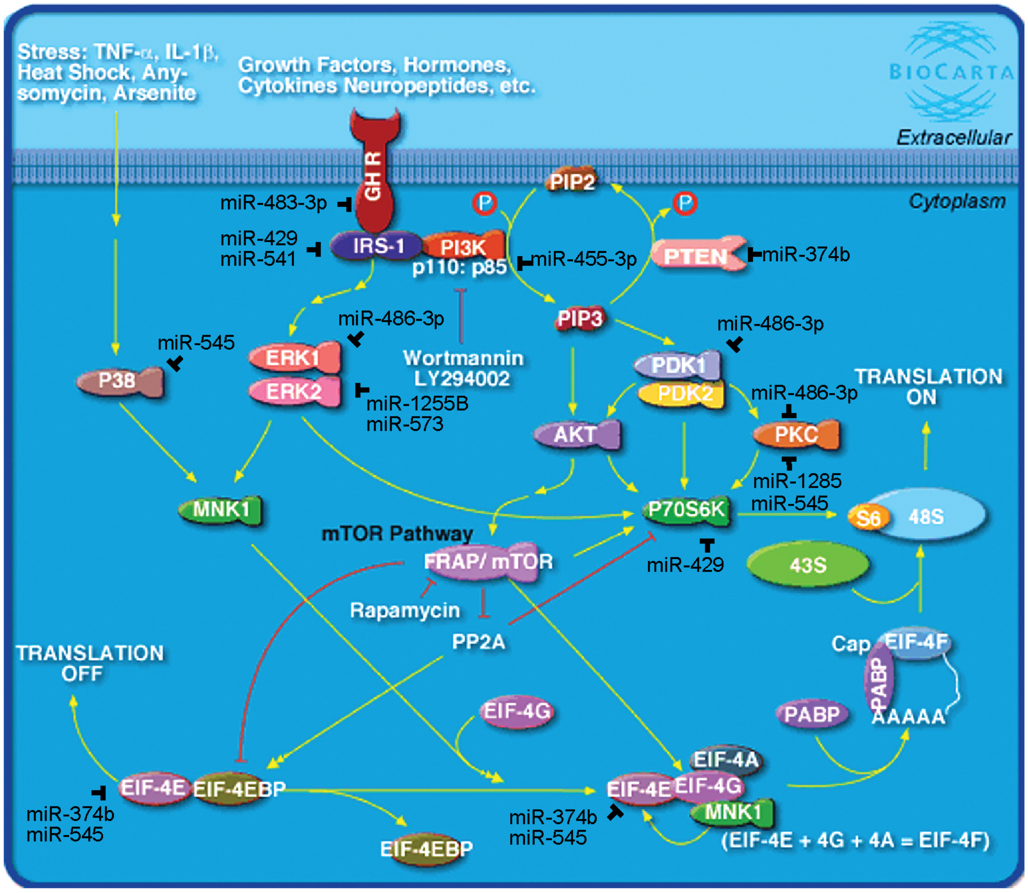

B. Relapse-free survival miRNA regulated pathway

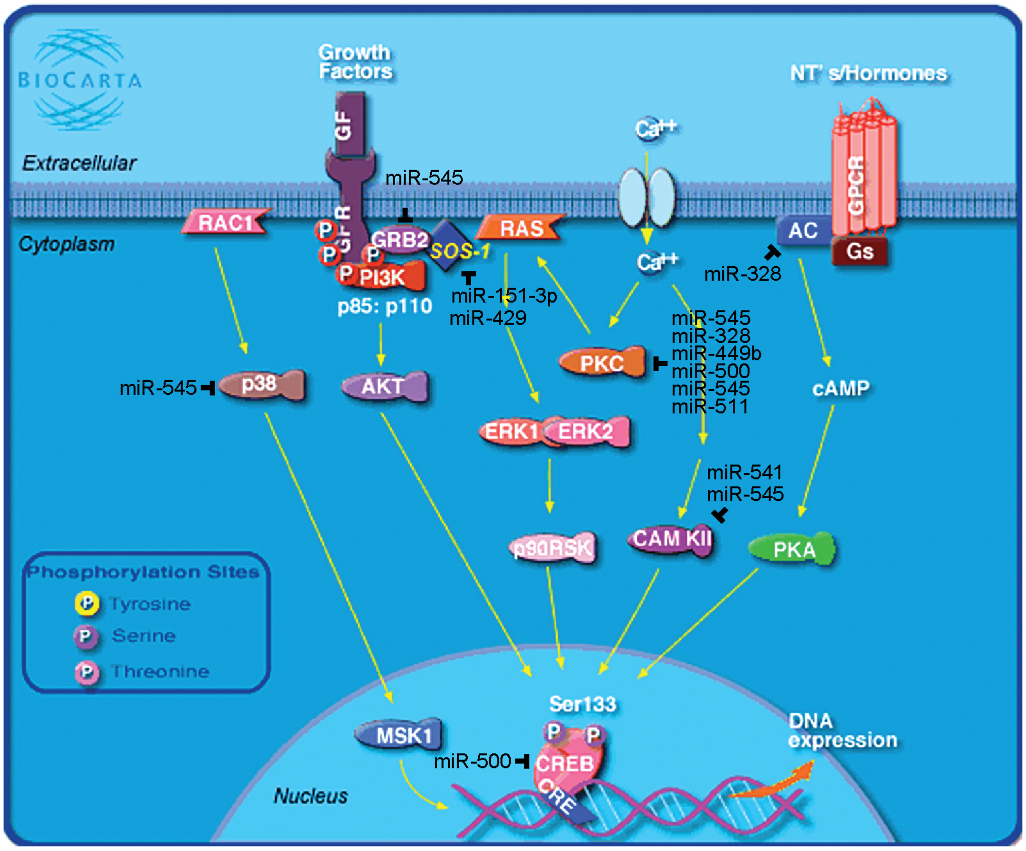

Supplementary Table S1.

Patient #	Anatomic site	Metastasis	Histology	Age at Dx	Gender	Race	Relapse	Dead
1	Right Distal Femur	No	Conventional	12	F	H	Yes	Yes
2	Right Distal Femur	No	Conventional	14	M	H	No	No
3	Left Femur	No	Conventional intramedullary	14	F	H	Yes	No
4	Right Distal Femur	No	Parosteal	14	M	H	No	No
5	Right Tibia	No	Conventional intramedullary	15.5	M	H	No	No
6	Left Femur	No	Conventional	16	F	H	Yes	No
7	Right Proximal Tibia	No	Conventional	16	M	H	No	No
8	Right Proximal Humerus	No	High grade surface	16	F	H	Yes (2nd primary)	No
9	Right Distal Femur	No	Conventional	17	F	WNH	Yes	Yes
10	Right Proximal Tibia	No	Conventional	18	M	Other	No	No
11	Left Proximal Tibia	No	Conventional	18	M	Black	Yes	Yes
12	Right Humerus	No	Conventional	19	M	WNH	Yes	No
13*	Right Leg - hip disarticulation	No	Conventional	19	M	H	No	No
14	Left Proximal Humerus	No	Parosteal	22	F	Other	No	No
15	Left Calf	No	Extraskeletal	28	M	H	No	No
16	Right Humerus	No	Conventional	28	M	WNH	Yes	Yes
17	Left Distal Femur	No	Conventional intramedullary	7	F	H	Yes	Yes
18	Right Distal Femur	No	Conventional	11	M	Black	Unknown	Yes
19	Right Proximal Tibia	No	Conventional intramedullary	16	F	H	No	Yes
20	Sacrum	No	Small cell osteosarcoma	19	F	H		Yes
21	Right Pelvis	No	Unknown	24	M	H	Yes	Yes
22	Right Distal Radius	No	Conventional	13	M	W	No	No
23	Right Distal Femur	No	Conventional	8	M	H	Yes	Yes
24	Right Proximal Tibia	No	High grade intramedullary with prominent surface	15	M	H	No	No
25	Right Proximal Tibia	No	Conventional	13	M	H	Yes	No
26	Left Proximal Tibia	No	Conventional	9	F	H	Yes (2nd primary)	Yes
27	Right Distal Femur	No	Conventional	21	F	H	Yes	Yes

*post 1 cycle of chemotherapy

Table S2. Overall survival associated miRNA pairs.

miR pairs	miRNA1			miRNA2		
	β	95% CI	p-value	β	95% CI	p-value
hsa-miR-221 & hsa-miR-1285	0.72	[0.34 - 1.11]	0.0003	-0.55	[-0.91 - -0.19]	0.003
hsa-miR-221 & hsa-miR-92a	0.70	[0.30 - 1.10]	0.0006	0.35	[0.14 - 0.55]	0.001
hsa-miR-486-3p & hsa-miR-541	0.39	[0.16 - 0.63]	0.001	0.42	[0.17 - 0.67]	0.0009
hsa-miR-21 & hsa-miR-126	0.93	[0.36 - 1.50]	0.001	-0.55	[-0.88 - -0.21]	0.001
hsa-miR-455-3p & hsa-miR-628-3p	0.96	[0.37 - 1.56]	0.001	0.32	[0.12 - 0.51]	0.001
hsa-miR-92a & hsa-miR-770-5p	0.30	[0.11 - 0.48]	0.002	0.63	[0.24 - 1.02]	0.002
hsa-miR-429 & hsa-miR-628-3p	-0.41	[-0.68 - -0.14]	0.003	0.31	[0.13 - 0.50]	0.001
hsa-miR-374b & hsa-miR-770-5p	0.33	[0.09 - 0.57]	0.007	0.82	[0.36 - 1.27]	0.0004
hsa-miR-202 & hsa-miR-770-5p	0.52	[0.14 - 0.90]	0.007	0.65	[0.28 - 1.03]	0.0007
hsa-miR-429 & hsa-miR-770-5p	-0.33	[-0.56 - -0.10]	0.005	0.64	[0.26 - 1.03]	0.001
hsa-miR-1255B & hsa-miR-541	0.57	[0.17 - 0.98]	0.005	0.32	[0.12 - 0.52]	0.002
hsa-miR-483-3p & hsa-miR-541	0.45	[0.15 - 0.75]	0.003	0.29	[0.10 - 0.48]	0.002
hsa-miR-92a & hsa-miR-628-3p	0.27	[0.09 - 0.45]	0.003	0.30	[0.09 - 0.50]	0.004
hsa-miR-202 & hsa-miR-216b	0.69	[0.22 - 1.16]	0.004	-0.42	[-0.71 - -0.14]	0.003
hsa-miR-202 & hsa-miR-573	0.61	[0.20 - 1.02]	0.003	0.73	[0.22 - 1.23]	0.005
hsa-miR-374b & hsa-miR-483-3p	0.31	[0.08 - 0.53]	0.009	0.55	[0.21 - 0.89]	0.002
hsa-miR-202 & hsa-miR-545#	0.59	[0.19 - 0.99]	0.004	0.67	[0.21 - 1.12]	0.004
hsa-miR-890 & hsa-miR-1285	0.27	[0.09 - 0.45]	0.004	-0.45	[-0.77 - -0.14]	0.005
hsa-miR-21 & hsa-miR-541	0.59	[0.16 - 1.02]	0.007	0.28	[0.09 - 0.46]	0.003
hsa-miR-1255B & hsa-miR-876-5p	0.54	[0.15 - 0.93]	0.006	0.25	[0.08 - 0.43]	0.004
hsa-miR-770-5p & hsa-miR-126	0.56	[0.19 - 0.92]	0.003	-0.43	[-0.76 - -0.11]	0.009
hsa-miR-429 & hsa-miR-483-3p	-0.33	[-0.56 - -0.11]	0.003	0.44	[0.11 - 0.76]	0.008
hsa-miR-483-3p & hsa-miR-126	0.39	[0.12 - 0.67]	0.006	-0.45	[-0.76 - -0.13]	0.006
hsa-miR-1285 & hsa-miR-505#	-0.37	[-0.65 - -0.09]	0.010	1.06	[0.36 - 1.77]	0.003
hsa-miR-30c & hsa-miR-21	0.29	[0.07 - 0.51]	0.009	0.75	[0.25 - 1.24]	0.003
hsa-miR-890 & hsa-miR-541	0.26	[0.06 - 0.45]	0.010	0.29	[0.10 - 0.49]	0.004
hsa-miR-505# & hsa-miR-126	0.99	[0.32 - 1.65]	0.004	-0.45	[-0.80 - -0.11]	0.010
hsa-miR-888 & hsa-miR-483-3p	-0.69	[-1.18 - -0.20]	0.006	0.38	[0.10 - 0.67]	0.008
hsa-miR-483-3p & hsa-miR-455-3p	0.38	[0.09 - 0.68]	0.010	0.87	[0.24 - 1.50]	0.007
hsa-miR-202 & hsa-miR-455-3p	0.49	[0.12 - 0.87]	0.010	0.97	[0.25 - 1.68]	0.008

Table S3. Relapse-free survival associated miRNA pairs.

miR pairs	miRNA1			miRNA2		
	β	95% CI	p-value	β	95% CI	p-value
hsa-miR-151-3p & hsa-miR-483-3p	-0.27	[-0.45 - -0.10]	0.002	0.61	[0.26 - 0.96]	0.0005
hsa-miR-429 & hsa-miR-483-3p	-0.58	[-0.93 - -0.23]	0.001	0.70	[0.28 - 1.11]	0.001
hsa-miR-151-3p & hsa-miR-30b	-0.27	[-0.44 - -0.10]	0.002	0.76	[0.31 - 1.21]	0.001
hsa-miR-429 & hsa-miR-30b	-0.49	[-0.79 - -0.18]	0.002	0.72	[0.24 - 1.21]	0.004
hsa-miR-429 & hsa-miR-770-5p	-0.47	[-0.78 - -0.17]	0.002	0.73	[0.22 - 1.24]	0.005
hsa-miR-483-3p & hsa-miR-541	0.51	[0.19 - 0.84]	0.002	0.31	[0.09 - 0.53]	0.005
hsa-miR-30a-3p & hsa-miR-483-3p	0.42	[0.12 - 0.72]	0.006	0.51	[0.19 - 0.83]	0.002
hsa-miR-888 & hsa-miR-483-3p	-1.28	[-2.14 - -0.42]	0.004	0.57	[0.18 - 0.95]	0.004
hsa-miR-30b & hsa-miR-541	0.65	[0.22 - 1.07]	0.003	0.31	[0.09 - 0.53]	0.005
hsa-miR-30a-3p & hsa-miR-30b	0.40	[0.11 - 0.70]	0.008	0.63	[0.21 - 1.04]	0.003
hsa-miR-151-3p & hsa-miR-770-5p	-0.23	[-0.38 - -0.07]	0.005	0.64	[0.19 - 1.09]	0.005
hsa-miR-335 & hsa-miR-483-3p	0.62	[0.15 - 1.08]	0.009	0.49	[0.16 - 0.82]	0.004
hsa-miR-888 & hsa-miR-30b	-1.03	[-1.79 - -0.28]	0.007	0.56	[0.15 - 0.98]	0.008

OV-miR Target Gene	num of OV-miRs	Regulatory OV-miRs
LCOR	8	miR-545,miR-656,miR-499-5p,miR-202,miR-374b,miR-216b,miR-429,miR-888
UBN2	8	miR-221,miR-545,miR-656,miR-21,miR-485-3p,miR-374b,miR-429,miR-888
ZDHHC21	8	miR-221,miR-656,miR-483-3p,miR-485-3p,miR-628-3p,miR-374b,miR-429,miR-888
ENAH	7	miR-545,miR-656,miR-622,miR-890,miR-374b,miR-429,miR-888
ABHD2	6	miR-221,miR-573,miR-545,miR-622,miR-486-3p,miR-888
HIPK2	6	miR-1282,miR-1255B,miR-483-3p,miR-30c,miR-216b,miR-1285
INO80D	6	miR-770-5p,miR-545,miR-622,miR-485-3p,miR-30c,miR-888
NSL1	6	miR-221,miR-545,miR-656,miR-628-3p,miR-541,miR-486-3p
PHC3	6	miR-656,miR-499-5p,miR-485-3p,miR-890,miR-374b,miR-429
UNC5C	6	miR-221,miR-770-5p,miR-573,miR-545,miR-499-5p,miR-541
YOD1	6	miR-21,miR-485-3p,miR-30c,miR-374b,miR-429,miR-888
ZBTB44	6	miR-545,miR-30c,miR-890,miR-216b,miR-1285,miR-888
ZNF704	6	miR-221,miR-656,miR-622,miR-890,miR-374b,miR-1285
GJA3	5	miR-92a,miR-374b,miR-429,miR-363,miR-888
JHDM1D	5	miR-656,miR-499-5p,miR-890,miR-374b,miR-429
N4BP2	5	miR-485-3p,miR-30c,miR-374b,miR-429,miR-888
NUFIP2	5	miR-221,miR-545,miR-656,miR-499-5p,miR-622
PPM1A	5	miR-656,miR-622,miR-374b,miR-429,miR-888
PRKAA2	5	miR-656,miR-499-5p,miR-92a,miR-30c,miR-363
PURB	5	miR-545,miR-499-5p,miR-890,miR-216b,miR-888
RC3H1	5	miR-545,miR-656,miR-628-3p,miR-374b,miR-888
RIMS3	5	miR-455-3p,miR-221,miR-545,miR-1255B,miR-1285
RRP15	5	miR-656,miR-374b,miR-1285,miR-429,miR-888
SLC5A3	5	miR-545,miR-656,miR-485-3p,miR-30c,miR-429
ST8SIA3	5	miR-656,miR-499-5p,miR-486-3p,miR-1285,miR-888
TAOK1	5	miR-221,miR-545,miR-30c,miR-374b,miR-876-5p
TMEM170B	5	miR-545,miR-30c,miR-890,miR-429,miR-888
ABL2	4	miR-505,miR-656,miR-30c,miR-888
ALG10B	4	miR-656,miR-374b,miR-429,miR-888
ATXN1	4	miR-221,miR-92a,miR-429,miR-363
CD84	4	miR-656,miR-1255B,miR-1285,miR-429
CYP20A1	4	miR-656,miR-890,miR-1285,miR-888

DCP2	4	miR-499-5p,miR-92a,miR-363,miR-888
DDHD1	4	miR-770-5p,miR-545,miR-622,miR-890
DOK6	4	miR-545,miR-656,miR-485-3p,miR-374b
KCNQ3	4	miR-221,miR-216b,miR-1285,miR-888
LYRM2	4	miR-455-3p,miR-573,miR-499-5p,miR-429
MKLN1	4	miR-656,miR-1255B,miR-890,miR-429
MRPL19	4	miR-545,miR-485-3p,miR-30c,miR-888
NFIB	4	miR-455-3p,miR-30c,miR-374b,miR-429
PLCXD3	4	miR-221,miR-656,miR-622,miR-374b
PLEKHA6	4	miR-221,miR-622,miR-541,miR-1285
SAMD8	4	miR-656,miR-622,miR-374b,miR-888
SC5DL	4	miR-545,miR-656,miR-374b,miR-1285
SLC7A11	4	miR-656,miR-30c,miR-374b,miR-429
SNTB2	4	miR-374b,miR-1285,miR-429,miR-888
SPRY3	4	miR-455-3p,miR-656,miR-890,miR-216b
SSR1	4	miR-545,miR-656,miR-1255B,miR-485-3p
TMEM135	4	miR-545,miR-483-3p,miR-374b,miR-429
TRPS1	4	miR-455-3p,miR-770-5p,miR-890,miR-374b
VAPB	4	miR-221,miR-483-3p,miR-485-3p,miR-486-3p
XPO4	4	miR-545,miR-656,miR-499-5p,miR-888
ZEB2	4	miR-656,miR-92a,miR-429,miR-363
ZNF780B	4	miR-656,miR-485-3p,miR-890,miR-1285
ADAMTS5	3	miR-656,miR-485-3p,miR-888
ANTXR2	3	miR-770-5p,miR-499-5p,miR-622
APOL6	3	miR-545,miR-499-5p,miR-1285
C11orf41	3	miR-455-3p,miR-221,miR-483-3p
C9orf5	3	miR-628-3p,miR-374b,miR-888
CBL	3	miR-545,miR-374b,miR-429
CREBZF	3	miR-221,miR-545,miR-499-5p
DSC3	3	miR-656,miR-429,miR-888
EEA1	3	miR-656,miR-30c,miR-374b
FGD4	3	miR-545,miR-499-5p,miR-374b
FGD6	3	miR-656,miR-374b,miR-429

FRS2	3	miR-221,miR-374b,miR-429
FXR1	3	miR-455-3p,miR-499-5p,miR-30c
GGCX	3	miR-221,miR-541,miR-890
GRIN2A	3	miR-656,miR-1285,miR-429
HUNK	3	miR-455-3p,miR-545,miR-890
KCNH1	3	miR-221,miR-486-3p,miR-1285
KCTD12	3	miR-374b,miR-429,miR-888
KIAA0408	3	miR-656,miR-374b,miR-429
KIAA1549	3	miR-545,miR-499-5p,miR-1255B
LRRC27	3	miR-374b,miR-1285,miR-888
LSAMP	3	miR-455-3p,miR-656,miR-890
MBLAC2	3	miR-656,miR-374b,miR-429
MED12L	3	miR-545,miR-656,miR-30c
METTL8	3	miR-545,miR-541,miR-429
MIPOL1	3	miR-656,miR-374b,miR-429
MTMR9	3	miR-545,miR-656,miR-429
NEDD4L	3	miR-545,miR-656,miR-890
PAPD5	3	miR-656,miR-429,miR-888
PCGF3	3	miR-545,miR-485-3p,miR-486-3p
PDE10A	3	miR-545,miR-485-3p,miR-374b
PGM2L1	3	miR-545,miR-656,miR-374b
QKI	3	miR-656,miR-374b,miR-429
RAB11FIP2	3	miR-545,miR-656,miR-429
RANBP10	3	miR-541,miR-429,miR-888
SAR1B	3	miR-656,miR-1285,miR-888
SEC23IP	3	miR-545,miR-485-3p,miR-888
SECISBP2L	3	miR-656,miR-499-5p,miR-429
SLC4A4	3	miR-374b,miR-429,miR-888
SPRED1	3	miR-770-5p,miR-374b,miR-888
STEAP2	3	miR-656,miR-485-3p,miR-374b
STK38L	3	miR-656,miR-374b,miR-888
TC2N	3	miR-656,miR-30c,miR-429
THRB	3	miR-890,miR-1285,miR-429

TRIM33	3	miR-545,miR-429,miR-888
TSHZ2	3	miR-545,miR-1255B,miR-30c
USP46	3	miR-30c,miR-374b,miR-429
ZBTB41	3	miR-656,miR-30c,miR-374b
ZNF322A	3	miR-656,miR-485-3p,miR-1285
ZNF714	3	miR-770-5p,miR-656,miR-374b
ZNF805	3	miR-545,miR-30c,miR-888
ZNF814	3	miR-221,miR-890,miR-1285
ABCE1	2	miR-656,miR-374b
ACOT13	2	miR-890,miR-1285
ACSL6	2	miR-656,miR-374b
ADAM19	2	miR-545,miR-1285
ADAT2	2	miR-545,miR-1285
AGFG1	2	miR-221,miR-429
AKAP13	2	miR-890,miR-1285
AKAP5	2	miR-656,miR-1255B
ALS2CR8	2	miR-656,miR-429
ARGFX	2	miR-656,miR-1285
ARHGAP26	2	miR-545,miR-890
ARHGAP42	2	miR-545,miR-656
ARID5B	2	miR-1285,miR-888
ARRB1	2	miR-455-3p,miR-545
ATP11A	2	miR-545,miR-1255B
ATP7A	2	miR-499-5p,miR-888
ATRX	2	miR-628-3p,miR-888
ATXN3	2	miR-656,miR-374b
B4GALT6	2	miR-656,miR-429
BACH2	2	miR-545,miR-1285
BCAT1	2	miR-545,miR-499-5p
C17orf51	2	miR-221,miR-545
C18orf63	2	miR-656,miR-374b
C1QTNF7	2	miR-656,miR-890
C20orf177	2	miR-545,miR-429

C5orf24	2	miR-374b,miR-429
C6orf89	2	miR-221,miR-30c
CBFA2T2	2	miR-455-3p,miR-1285
CCDC68	2	miR-485-3p,miR-374b
CCNJ	2	miR-622,miR-429
CDC73	2	miR-374b,miR-429
CELF2	2	miR-545,miR-888
CEP44	2	miR-656,miR-1285
CERS6	2	miR-656,miR-374b
CHM	2	miR-374b,miR-429
CHML	2	miR-656,miR-429
CLCC1	2	miR-1285,miR-429
CNOT6L	2	miR-429,miR-888
CPM	2	miR-656,miR-499-5p
CPSF6	2	miR-545,miR-30c
CSRNP3	2	miR-545,miR-656
CXorf23	2	miR-499-5p,miR-374b
CYP1B1	2	miR-656,miR-429
DBT	2	miR-221,miR-216b
DCBLD2	2	miR-656,miR-429
DLG2	2	miR-485-3p,miR-374b
DMD	2	miR-656,miR-374b
DNAJC3	2	miR-1285,miR-429
DZIP1	2	miR-890,miR-429
EIF2C4	2	miR-455-3p,miR-545
EIF4E	2	miR-545,miR-374b
EIF4E3	2	miR-545,miR-656
EIF5A2	2	miR-656,miR-374b
ELK4	2	miR-485-3p,miR-429
ELOVL7	2	miR-656,miR-374b
ENTPD7	2	miR-221,miR-545
EPM2AIP1	2	miR-499-5p,miR-429
FAM124A	2	miR-485-3p,miR-1285

FAM126B	2	miR-429,miR-888
FAM13A	2	miR-545,miR-485-3p
FAM190B	2	miR-545,miR-656
FAM46A	2	miR-221,miR-876-5p
FAM60A	2	miR-656,miR-628-3p
FEM1C	2	miR-656,miR-374b
FGF2	2	miR-545,miR-499-5p
FOXK1	2	miR-545,miR-890
FOXN2	2	miR-499-5p,miR-888
FRAS1	2	miR-622,miR-429
GABRA1	2	miR-221,miR-374b
GDAP1	2	miR-499-5p,miR-888
GFPT1	2	miR-656,miR-429
GLDN	2	miR-545,miR-888
GMFB	2	miR-770-5p,miR-429
GNRHR	2	miR-656,miR-374b
GOLGA7B	2	miR-485-3p,miR-1285
GPC6	2	miR-656,miR-429
GXYLT1	2	miR-429,miR-888
HBS1L	2	miR-374b,miR-429
HCFC2	2	miR-656,miR-485-3p
HNRNPA3	2	miR-545,miR-30c
ICK	2	miR-656,miR-374b
IDS	2	miR-656,miR-374b
IL6ST	2	miR-485-3p,miR-374b
IMPG2	2	miR-656,miR-499-5p
IPO7	2	miR-374b,miR-429
ITPRIPL2	2	miR-770-5p,miR-656
JMY	2	miR-656,miR-374b
KATNAL1	2	miR-890,miR-429
KIAA0319	2	miR-455-3p,miR-30c
KIAA1456	2	miR-545,miR-374b
KIAA1462	2	miR-1285,miR-429

LDOC1L	2	miR-455-3p,miR-1285
LHFPL2	2	miR-374b,miR-888
LIN7C	2	miR-499-5p,miR-374b
LRRC19	2	miR-656,miR-374b
LRRC8C	2	miR-374b,miR-888
MAP3K13	2	miR-656,miR-1285
MAP4K5	2	miR-429,miR-888
MAP9	2	miR-656,miR-30c
MAPK1IP1L	2	miR-656,miR-374b
MBL2	2	miR-656,miR-499-5p
MDM2	2	miR-656,miR-374b
MED13	2	miR-374b,miR-429
MFAP3L	2	miR-455-3p,miR-1255B
MIB1	2	miR-374b,miR-888
MKI67	2	miR-545,miR-1255B
MLEC	2	miR-541,miR-888
MOBKL1A	2	miR-656,miR-890
MTX3	2	miR-545,miR-890
MYBL1	2	miR-221,miR-656
MYEF2	2	miR-656,miR-499-5p
MYO1B	2	miR-656,miR-485-3p
MYO5B	2	miR-545,miR-374b
NCOA7	2	miR-499-5p,miR-374b
NEGR1	2	miR-656,miR-429
NF1	2	miR-374b,miR-429
NFYB	2	miR-485-3p,miR-888
ODZ1	2	miR-656,miR-485-3p
ODZ4	2	miR-455-3p,miR-1285
OSBPL3	2	miR-221,miR-656
OTUD7B	2	miR-455-3p,miR-429
PAK2	2	miR-374b,miR-888
PARP8	2	miR-656,miR-374b
PCGF5	2	miR-656,miR-888

PCNX	2	miR-374b,miR-429
PEG10	2	miR-545,miR-30c
PELI2	2	miR-656,miR-429
PHTF2	2	miR-656,miR-30c
PLA2G12A	2	miR-545,miR-656
PLCB1	2	miR-656,miR-1285
PLCXD1	2	miR-890,miR-1285
PLD5	2	miR-656,miR-374b
POLK	2	miR-374b,miR-429
POTEM	2	miR-545,miR-1285
PRKCA	2	miR-545,miR-1285
PRPF4B	2	miR-545,miR-656
PRRC1	2	miR-656,miR-374b
RAB11A	2	miR-374b,miR-888
RAP2B	2	miR-656,miR-890
RBAK	2	miR-374b,miR-888
RBMS2	2	miR-545,miR-1285
REEP1	2	miR-545,miR-656
RMND5A	2	miR-455-3p,miR-374b
ROBO2	2	miR-622,miR-888
ROCK2	2	miR-622,miR-429
ROD1	2	miR-221,miR-545
SAMD4A	2	miR-485-3p,miR-888
SARM1	2	miR-545,miR-1285
SCAMP1	2	miR-656,miR-429
SEC62	2	miR-221,miR-656
SEC63	2	miR-656,miR-374b
SEMA3D	2	miR-374b,miR-888
SEMA5A	2	miR-374b,miR-429
SESTD1	2	miR-485-3p,miR-374b
SGPP1	2	miR-656,miR-374b
SGTB	2	miR-656,miR-890
SHROOM4	2	miR-1285,miR-429

SIK3	2	miR-656,miR-374b
SLC26A9	2	miR-890,miR-1285
SLC2A13	2	miR-221,miR-656
SLC35B4	2	miR-30c,miR-429
SLC35E2	2	miR-545,miR-1285
SLC35F1	2	miR-455-3p,miR-656
SLC4A7	2	miR-221,miR-429
SMCR7L	2	miR-545,miR-429
SMU1	2	miR-545,miR-890
SMURF1	2	miR-545,miR-1285
SOS1	2	miR-374b,miR-429
SPOPL	2	miR-656,miR-374b
SRSF1	2	miR-622,miR-429
STAM2	2	miR-374b,miR-429
STAMBP	2	miR-545,miR-1285
STEAP4	2	miR-656,miR-374b
STXBP4	2	miR-656,miR-485-3p
STYX	2	miR-374b,miR-429
SUPT7L	2	miR-499-5p,miR-1255B
TCF4	2	miR-485-3p,miR-374b
TFDP2	2	miR-890,miR-1285
THAP2	2	miR-656,miR-429
TMEM215	2	miR-545,miR-485-3p
TMEM237	2	miR-545,miR-888
TMEM87B	2	miR-656,miR-30c
TMX1	2	miR-890,miR-429
TPD52	2	miR-656,miR-374b
TRAM2	2	miR-890,miR-888
TRDMT1	2	miR-221,miR-216b
TRERF1	2	miR-455-3p,miR-545
TRPM6	2	miR-656,miR-374b
TRPM7	2	miR-1285,miR-888
TTC33	2	miR-656,miR-374b

TTC39C	2	miR-545,miR-890
TXLNB	2	miR-656,miR-374b
UBA6	2	miR-485-3p,miR-374b
UCHL5	2	miR-485-3p,miR-429
UHMK1	2	miR-545,miR-374b
USP12	2	miR-656,miR-374b
USP31	2	miR-545,miR-429
VAMP4	2	miR-656,miR-374b
VAPA	2	miR-656,miR-499-5p
WDR17	2	miR-656,miR-374b
WNK3	2	miR-545,miR-429
XRN1	2	miR-455-3p,miR-888
YIPF6	2	miR-499-5p,miR-30c
ZBTB46	2	miR-545,miR-374b
ZC3HAV1	2	miR-545,miR-1285
ZDHHC20	2	miR-656,miR-30c
ZNF117	2	miR-770-5p,miR-656
ZNF24	2	miR-545,miR-374b
AADACL3	1	miR-545
ABCA5	1	miR-656
ABCC5	1	miR-545
ACAD8	1	miR-455-3p
ACPL2	1	miR-545
ADCYAP1	1	miR-485-3p
AEBP2	1	miR-656
AGPAT5	1	miR-656
AHR	1	miR-656
AIF1L	1	miR-455-3p
AKAP11	1	miR-656
AKT3	1	miR-455-3p
ALG13	1	miR-656
AMPD3	1	miR-890
ANGPTL5	1	miR-656

ANKRD40	1	miR-499-5p
ANKS6	1	miR-890
ANO3	1	miR-656
AP3M2	1	miR-455-3p
APOOL	1	miR-429
ARFGEF2	1	miR-656
ARHGAP20	1	miR-656
ARHGAP28	1	miR-656
ARHGAP29	1	miR-656
ARHGEF12	1	miR-888
ARHGEF6	1	miR-545
ARID2	1	miR-656
ARIH1	1	miR-545
ARIH2	1	miR-429
ARL13B	1	miR-656
ARL4A	1	miR-545
ARL5A	1	miR-429
ARNT	1	miR-545
ARPP19	1	miR-656
ARRDC3	1	miR-545
ASPH	1	miR-656
ATG12	1	miR-656
ATP11B	1	miR-429
ATP2C1	1	miR-888
ATXN1L	1	miR-545
ATXN7L1	1	miR-545
B3GALTL	1	miR-429
BAG4	1	miR-656
BANP	1	miR-545
BCL2L11	1	miR-429
BCL2L15	1	miR-545
BDP1	1	miR-429
BEND2	1	miR-656

BEND3	1	miR-455-3p
BET1L	1	miR-545
BTLA	1	miR-656
BVES	1	miR-545
BZW1	1	miR-656
C10orf47	1	miR-499-5p
C11orf58	1	miR-890
C11orf87	1	miR-656
C11orf95	1	miR-429
C13orf30	1	miR-656
C13orf31	1	miR-888
C14orf126	1	miR-656
C15orf29	1	miR-485-3p
C16orf52	1	miR-429
C16orf72	1	miR-429
C17orf75	1	miR-499-5p
C18orf25	1	miR-656
C19orf12	1	miR-429
C1QTNF3	1	miR-656
C1RL	1	miR-455-3p
C1orf115	1	miR-545
C1orf96	1	miR-429
C21orf91	1	miR-429
C3orf64	1	miR-221
C4orf32	1	miR-656
C5orf47	1	miR-656
C6	1	miR-221
C6orf120	1	miR-429
C6orf35	1	miR-545
C6orf97	1	miR-656
C7orf57	1	miR-656
C7orf58	1	miR-429
C9orf91	1	miR-890

CAB39L	1	miR-455-3p
CACNA1C	1	miR-545
CADM1	1	miR-545
CALCRL	1	miR-545
CAMK2D	1	miR-545
CAMSAP1	1	miR-485-3p
CAMSAP1L1	1	miR-429
CAPRIN1	1	miR-656
CASC4	1	miR-429
CASK	1	miR-499-5p
CAV2	1	miR-656
CCBE1	1	miR-656
CCDC144A	1	miR-545
CCDC47	1	miR-888
CCDC85C	1	miR-455-3p
CCDC88A	1	miR-656
CCPG1	1	miR-545
CD109	1	miR-656
CD28	1	miR-888
CD99L2	1	miR-455-3p
CDC27	1	miR-656
CDC42SE2	1	miR-545
CDK12	1	miR-656
CDK19	1	miR-221
CENPK	1	miR-656
CEP135	1	miR-656
CERS3	1	miR-545
CFL2	1	miR-429
CGGBP1	1	miR-485-3p
CHN2	1	miR-545
CHP	1	miR-890
CHRNB2	1	miR-890
CHURC1	1	miR-656

CLDN1	1	miR-888
CLDN18	1	miR-888
CLEC5A	1	miR-888
CMTM4	1	miR-545
CNTN4	1	miR-656
COA5	1	miR-890
COL4A3BP	1	miR-429
COX11	1	miR-429
CPD	1	miR-485-3p
CPPED1	1	miR-545
CREB1	1	miR-656
CREBL2	1	miR-656
CSNK1G3	1	miR-545
CTDSPL2	1	miR-656
DARS2	1	miR-656
DAZ1	1	miR-429
DAZ2	1	miR-429
DAZ3	1	miR-429
DAZ4	1	miR-429
DCAF17	1	miR-545
DCAF7	1	miR-455-3p
DERL1	1	miR-888
DGCR14	1	miR-545
DGKB	1	miR-656
DIRAS2	1	miR-429
DNA2	1	miR-429
DNAJC11	1	miR-545
DNAJC21	1	miR-656
DNM1L	1	miR-485-3p
DNM3	1	miR-545
DPH3	1	miR-545
DPP10	1	miR-888
DPY19L1	1	miR-545

DPYD	1	miR-656
DRP2	1	miR-429
DTWD2	1	miR-656
EDNRA	1	miR-429
EDNRB	1	miR-656
EIF2S3	1	miR-221
ELAVL2	1	miR-485-3p
ELF3	1	miR-455-3p
ELF4	1	miR-545
ELMOD2	1	miR-429
EML6	1	miR-221
ENPP1	1	miR-545
EPHA7	1	miR-888
ERCC4	1	miR-499-5p
ERG	1	miR-499-5p
ERI1	1	miR-429
ERMN	1	miR-888
ERO1LB	1	miR-656
ERP44	1	miR-656
ERRFI1	1	miR-429
ESRRG	1	miR-429
ETV1	1	miR-545
EVI2A	1	miR-890
EVI5	1	miR-890
EXOC8	1	miR-656
EYA4	1	miR-656
FAM110C	1	miR-545
FAM117B	1	miR-656
FAM126A	1	miR-656
FAM168B	1	miR-888
FAM169A	1	miR-429
FAM198B	1	miR-656
FAM46C	1	miR-888

FAM76A	1	miR-656
FAM8A1	1	miR-429
FBXL3	1	miR-656
FBXO28	1	miR-656
FBXO44	1	miR-455-3p
FBXO9	1	miR-888
FGF1	1	miR-485-3p
FGF9	1	miR-499-5p
FGFR1	1	miR-545
FGL2	1	miR-656
FLRT2	1	miR-545
FNDC3B	1	miR-429
FNDC5	1	miR-455-3p
FRYL	1	miR-656
FSD1L	1	miR-429
FUT4	1	miR-429
G3BP2	1	miR-888
GAB1	1	miR-429
GABRB3	1	miR-656
GAPVD1	1	miR-656
GBP4	1	miR-545
GIGYF2	1	miR-455-3p
GNAI3	1	miR-545
GNAT1	1	miR-656
GNE	1	miR-888
GNL1	1	miR-545
GOLGA1	1	miR-221
GPCPD1	1	miR-656
GPD1	1	miR-545
GPD2	1	miR-545
GPM6A	1	miR-429
GPR12	1	miR-656
GPR85	1	miR-455-3p

GRB2	1	miR-545
GRIA2	1	miR-656
GRSF1	1	miR-545
GTPBP1	1	miR-455-3p
HAUS2	1	miR-545
HAUS6	1	miR-656
HCN1	1	miR-485-3p
HEATR1	1	miR-656
HEG1	1	miR-429
HIGD1A	1	miR-545
HNF4G	1	miR-656
HOXA3	1	miR-455-3p
HSF5	1	miR-656
ICA1L	1	miR-429
IER2	1	miR-455-3p
IFNAR1	1	miR-545
IFRD1	1	miR-221
IGF1	1	miR-656
IKZF1	1	miR-888
IL10RA	1	miR-545
IL20RA	1	miR-656
IL4R	1	miR-545
ILDR2	1	miR-545
IMPA1	1	miR-656
IMPAD1	1	miR-656
IPMK	1	miR-429
IPPK	1	miR-545
IRF4	1	miR-545
ITGB1	1	miR-888
JPH1	1	miR-545
KAT2B	1	miR-656
KBTBD6	1	miR-429
KCNIP4	1	miR-656

KCNJ2	1	miR-455-3p
KCNQ5	1	miR-485-3p
KCTD20	1	miR-888
KCTD8	1	miR-429
KIAA1430	1	miR-429
KIAA1432	1	miR-429
KIAA1486	1	miR-656
KIAA1737	1	miR-545
KIF1B	1	miR-545
KIF3A	1	miR-888
KL	1	miR-656
KLF4	1	miR-656
KLF8	1	miR-656
KLHL4	1	miR-656
KMO	1	miR-656
KRIT1	1	miR-656
L2HGDH	1	miR-888
LBR	1	miR-656
LCA5	1	miR-656
LGSN	1	miR-656
LIMA1	1	miR-499-5p
LOC401097	1	miR-656
LPAR1	1	miR-656
LPCAT2	1	miR-656
LPPR5	1	miR-656
LRP8	1	miR-888
LRRC31	1	miR-656
LRRCC1	1	miR-221
LTN1	1	miR-429
LYRM7	1	miR-656
MANEA	1	miR-656
MAPK14	1	miR-545
MARCH5	1	miR-485-3p

MARK1	1	miR-221
MASP1	1	miR-485-3p
MBOAT2	1	miR-545
MCC	1	miR-455-3p
MCFD2	1	miR-429
MDFIC	1	miR-656
MED20	1	miR-545
MEOX1	1	miR-455-3p
METTL16	1	miR-545
MFAP5	1	miR-545
MICB	1	miR-656
MMS22L	1	miR-429
MRO	1	miR-545
MRP63	1	miR-656
MRPL42	1	miR-656
MSN	1	miR-429
MTAP	1	miR-429
MUTED	1	miR-545
MYCT1	1	miR-890
MYLK	1	miR-545
MYNN	1	miR-888
MYO3B	1	miR-656
MYOT	1	miR-656
MYOZ2	1	miR-656
NAA25	1	miR-656
NANOS1	1	miR-429
NCOA3	1	miR-429
NECAB1	1	miR-429
NEK4	1	miR-656
NEK7	1	miR-656
NEMF	1	miR-656
NHLRC2	1	miR-656
NIPA1	1	miR-656

NKPD1	1	miR-455-3p
NLGN4X	1	miR-429
NOM1	1	miR-485-3p
NOS1AP	1	miR-545
NOTCH2	1	miR-656
NR1D2	1	miR-656
NR5A2	1	miR-429
NRAS	1	miR-888
NRIP1	1	miR-656
NRIP3	1	miR-890
NRXN1	1	miR-890
NXT2	1	miR-656
OCIAD1	1	miR-656
OMG	1	miR-499-5p
OSTM1	1	miR-429
OTUD1	1	miR-545
PABPC4L	1	miR-656
PAIP2B	1	miR-499-5p
PCDH11Y	1	miR-656
PCDH19	1	miR-545
PCSK1	1	miR-656
PCYT1B	1	miR-890
PDCD4	1	miR-499-5p
PDE5A	1	miR-429
PDGFB	1	miR-455-3p
PDXDC1	1	miR-545
PEX19	1	miR-888
PHKB	1	miR-656
PIGM	1	miR-429
PIK3AP1	1	miR-545
PIK3C2A	1	miR-656
PIK3R1	1	miR-455-3p
PKD2	1	miR-888

PLA2G15	1	miR-455-3p
PLEKHB2	1	miR-545
PLXDC1	1	miR-545
PMPCB	1	miR-429
POF1B	1	miR-656
POM121	1	miR-485-3p
POM121C	1	miR-485-3p
PPIE	1	miR-545
PPM1K	1	miR-485-3p
PPP1CB	1	miR-429
PPP1R13B	1	miR-455-3p
PPP1R15B	1	miR-656
PPP2R1B	1	miR-429
PRKAA1	1	miR-656
PRKAB1	1	miR-455-3p
PRKCI	1	miR-656
PRKX	1	miR-545
PRPF40A	1	miR-485-3p
PSEN1	1	miR-545
PTCH1	1	miR-545
PTER	1	miR-656
PTPRB	1	miR-890
PUM2	1	miR-485-3p
PURA	1	miR-656
PYROXD1	1	miR-656
QSER1	1	miR-656
RAB10	1	miR-545
RAB27B	1	miR-888
RAB33B	1	miR-429
RAB3D	1	miR-455-3p
RACGAP1	1	miR-656
RALGAPB	1	miR-485-3p
RAP2A	1	miR-485-3p

RASGRP3	1	miR-888
RBBP9	1	miR-545
RBM20	1	miR-545
RBM8A	1	miR-429
RBMS1	1	miR-485-3p
RCN2	1	miR-888
RCOR3	1	miR-656
RECK	1	miR-429
REPS2	1	miR-656
RFX7	1	miR-656
RGS6	1	miR-221
RHBDD1	1	miR-499-5p
RHOBTB3	1	miR-656
RIF1	1	miR-545
RIMKLB	1	miR-656
RIMS1	1	miR-499-5p
RNF169	1	miR-499-5p
RNF182	1	miR-888
RNF19A	1	miR-429
RNF2	1	miR-429
RPS23	1	miR-429
RPS6KA2	1	miR-545
RPS6KB1	1	miR-429
RUNX1T1	1	miR-221
SAMD5	1	miR-429
SASH1	1	miR-656
SASS6	1	miR-656
SDPR	1	miR-656
SEL1L	1	miR-656
SEMA3A	1	miR-499-5p
SEMA4F	1	miR-888
SEPT2	1	miR-888
SERPINB8	1	miR-455-3p

SERTAD2	1	miR-888
SH3BGRL2	1	miR-656
SH3RF1	1	miR-656
SHC4	1	miR-429
SHISA2	1	miR-429
SKA2	1	miR-656
SKA3	1	miR-429
SKP1	1	miR-499-5p
SLC11A2	1	miR-656
SLC12A2	1	miR-888
SLC12A6	1	miR-888
SLC13A1	1	miR-656
SLC15A5	1	miR-429
SLC1A4	1	miR-545
SLC20A2	1	miR-656
SLC25A30	1	miR-656
SLC30A4	1	miR-499-5p
SLC35A5	1	miR-656
SLC35E1	1	miR-545
SLC35F5	1	miR-429
SLC38A1	1	miR-656
SLC41A2	1	miR-545
SLC5A1	1	miR-545
SLC5A12	1	miR-485-3p
SLCO1C1	1	miR-656
SLITRK4	1	miR-656
SMAD5	1	miR-429
SNAP25	1	miR-545
SNAP29	1	miR-545
SNRK	1	miR-499-5p
SOCS5	1	miR-888
SOCS6	1	miR-656
SOX4	1	miR-656

SOX6	1	miR-499-5p
SPAG9	1	miR-656
SPATA13	1	miR-485-3p
SPATA5	1	miR-656
SPDYE5	1	miR-545
SPIN4	1	miR-656
SRSF6	1	miR-888
SSH1	1	miR-485-3p
SSX2IP	1	miR-656
ST6GAL2	1	miR-485-3p
STAC2	1	miR-545
SUCLG2	1	miR-656
SUMO3	1	miR-545
SUSD5	1	miR-429
SYNJ1	1	miR-545
TAF9B	1	miR-545
TBC1D19	1	miR-656
TBC1D22A	1	miR-455-3p
TBC1D8B	1	miR-545
TBRG1	1	miR-656
TBX18	1	miR-429
TCEANC2	1	miR-545
TCL1A	1	miR-1282
TERF1	1	miR-429
TET2	1	miR-545
TFAP2A	1	miR-429
TFPI	1	miR-545
TGFBR1	1	miR-545
TGFBR3	1	miR-656
THBS2	1	miR-545
TIFA	1	miR-656
TLL2	1	miR-429
TMCC1	1	miR-888

TMED7	1	miR-429
TMEM108	1	miR-656
TMEM110	1	miR-545
TMEM159	1	miR-890
TMEM167A	1	miR-429
TMEM184B	1	miR-485-3p
TMEM185B	1	miR-545
TMEM194A	1	miR-545
TMEM26	1	miR-429
TMEM30A	1	miR-656
TMEM47	1	miR-499-5p
TMEM64	1	miR-656
TMOD3	1	miR-429
TMTC3	1	miR-888
TP53BP2	1	miR-221
TPM3	1	miR-888
TRAF6	1	miR-499-5p
TRHDE	1	miR-888
TRMT5	1	miR-429
TRPA1	1	miR-656
TSC1	1	miR-221
TSGA14	1	miR-429
TSPAN14	1	miR-455-3p
UBASH3B	1	miR-656
UBE2W	1	miR-656
UBFD1	1	miR-888
UNC80	1	miR-545
USP15	1	miR-545
USP9X	1	miR-545
UTY	1	miR-429
VASH2	1	miR-429
VAV3	1	miR-499-5p
VMA21	1	miR-888

VTI1A	1	miR-429
WAPAL	1	miR-429
WASF2	1	miR-890
WASF3	1	miR-429
WDFY1	1	miR-888
WDR82	1	miR-429
WEE1	1	miR-545
WIPF1	1	miR-429
XKR9	1	miR-888
ZBTB40	1	miR-455-3p
ZC3H12C	1	miR-656
ZDHHC15	1	miR-656
ZDHHC17	1	miR-429
ZDHHC23	1	miR-888
ZEB1	1	miR-429
ZFAND5	1	miR-221
ZFP30	1	miR-656
ZFPM2	1	miR-429
ZFX	1	miR-429
ZFY	1	miR-656
ZIC1	1	miR-545
ZIC5	1	miR-485-3p
ZKSCAN3	1	miR-890
ZMYM4	1	miR-485-3p
ZNF10	1	miR-656
ZNF100	1	miR-656
ZNF217	1	miR-499-5p
ZNF238	1	miR-455-3p
ZNF254	1	miR-656
ZNF260	1	miR-656
ZNF322B	1	miR-485-3p
ZNF417	1	miR-890
ZNF449	1	miR-656

ZNF470	1	miR-888
ZNF516	1	miR-429
ZNF532	1	miR-429
ZNF546	1	miR-656
ZNF569	1	miR-656
ZNF641	1	miR-888
ZNF670	1	miR-499-5p
ZNF687	1	miR-455-3p
ZNF793	1	miR-888
ZNF84	1	miR-656
ZNRF1	1	miR-545
ZSCAN23	1	miR-656
ZXDB	1	miR-221

RE-miR Target	num of RE-miRs	Regulatory RE-miRs
SH3TC2	12	miR-146b-5p,miR-500,miR-668,miR-30b,miR-221,miR-335,miR-499-5p,miR-483-3p,miR-30a-3p,miR-511,miR-429,miR-888
HOOK3	9	miR-500,miR-545,miR-30b,miR-499-5p,miR-30a-3p,miR-541,miR-511,miR-429,miR-888
AAK1	8	miR-500,miR-668,miR-30b,miR-335,miR-30a-3p,miR-511,miR-429,miR-888
KSR2	8	miR-500,miR-545,miR-335,miR-483-3p,miR-328,miR-541,miR-449b,miR-151-3p
HIPK2	6	miR-500,miR-668,miR-30b,miR-483-3p,miR-30a-3p,miR-511
KLF12	6	miR-500,miR-668,miR-30b,miR-499-5p,miR-30a-3p,miR-429
LOC100507421	6	miR-500,miR-545,miR-335,miR-499-5p,miR-483-3p,miR-541
NSL1	6	miR-500,miR-545,miR-668,miR-221,miR-541,miR-511
SLC1A2	6	miR-30b,miR-221,miR-335,miR-30a-3p,miR-511,miR-429
TMEM170B	6	miR-500,miR-545,miR-30b,miR-30a-3p,miR-429,miR-888
TNRC6B	6	miR-545,miR-30b,miR-335,miR-30a-3p,miR-449b,miR-888
CADM2	5	miR-500,miR-30b,miR-30a-3p,miR-511,miR-429
CLMN	5	miR-500,miR-335,miR-541,miR-511,miR-429
CSRNP3	5	miR-545,miR-668,miR-335,miR-449b,miR-511
DCUN1D5	5	miR-500,miR-545,miR-499-5p,miR-429,miR-888
LCOR	5	miR-545,miR-499-5p,miR-511,miR-429,miR-888
MDM4	5	miR-335,miR-770-5p,miR-541,miR-449b,miR-888
METTL8	5	miR-545,miR-335,miR-541,miR-511,miR-429
MRPL19	5	miR-500,miR-545,miR-30b,miR-30a-3p,miR-888
PRKAA2	5	miR-30b,miR-335,miR-499-5p,miR-30a-3p,miR-151-3p
PURB	5	miR-545,miR-335,miR-499-5p,miR-449b,miR-888
RANBP10	5	miR-328,miR-541,miR-449b,miR-429,miR-888
RC3H1	5	miR-500,miR-545,miR-151-3p,miR-511,miR-888
SLC5A3	5	miR-545,miR-30b,miR-30a-3p,miR-511,miR-429
STK35	5	miR-30b,miR-335,miR-30a-3p,miR-541,miR-449b
UBN2	5	miR-545,miR-221,miR-449b,miR-429,miR-888
ACVR2B	4	miR-545,miR-335,miR-483-3p,miR-449b
ALG10B	4	miR-500,miR-511,miR-429,miR-888
BCAT1	4	miR-500,miR-545,miR-499-5p,miR-511
C17orf51	4	miR-545,miR-221,miR-328,miR-449b
CACNA1E	4	miR-541,miR-449b,miR-511,miR-888
CCDC50	4	miR-30b,miR-30a-3p,miR-449b,miR-888
CDK6	4	miR-500,miR-335,miR-449b,miR-888
CDS2	4	miR-328,miR-449b,miR-429,miR-888
DCP2	4	miR-500,miR-335,miR-499-5p,miR-888
GATAD2B	4	miR-500,miR-668,miR-541,miR-449b
GPR180	4	miR-500,miR-545,miR-429,miR-888
IRGQ	4	miR-483-3p,miR-541,miR-449b,miR-511
LIN28B	4	miR-30b,miR-499-5p,miR-30a-3p,miR-429

LRPAP1	4	miR-545,miR-335,miR-541,miR-449b
N4BP2	4	miR-30b,miR-30a-3p,miR-429,miR-888
NFIB	4	miR-668,miR-30b,miR-30a-3p,miR-429
NXPH3	4	miR-545,miR-328,miR-541,miR-449b
PLEKHG4B	4	miR-328,miR-541,miR-449b,miR-511
PRKCA	4	miR-500,miR-545,miR-328,miR-449b
RIMKLA	4	miR-545,miR-335,miR-449b,miR-511
RRP15	4	miR-500,miR-151-3p,miR-429,miR-888
RTKN2	4	miR-30b,miR-335,miR-30a-3p,miR-429
SARM1	4	miR-500,miR-545,miR-328,miR-449b
SH3PXD2A	4	miR-30b,miR-30a-3p,miR-541,miR-449b
STX17	4	miR-545,miR-30b,miR-30a-3p,miR-429
TAOK1	4	miR-545,miR-30b,miR-221,miR-30a-3p
TMEM135	4	miR-545,miR-483-3p,miR-511,miR-429
TSHZ2	4	miR-545,miR-30b,miR-30a-3p,miR-449b
USP46	4	miR-30b,miR-30a-3p,miR-511,miR-429
YIPF6	4	miR-30b,miR-335,miR-499-5p,miR-30a-3p
YOD1	4	miR-30b,miR-30a-3p,miR-429,miR-888
ZBTB44	4	miR-545,miR-30b,miR-30a-3p,miR-888
ZDHHC21	4	miR-221,miR-483-3p,miR-429,miR-888
ZDHHC3	4	miR-545,miR-335,miR-541,miR-511
ZNF264	4	miR-545,miR-30b,miR-335,miR-30a-3p
ZNF805	4	miR-545,miR-30b,miR-30a-3p,miR-888
A1CF	3	miR-30b,miR-30a-3p,miR-511
ABHD2	3	miR-545,miR-221,miR-888
AP3S2	3	miR-541,miR-449b,miR-511
ATG2B	3	miR-30b,miR-30a-3p,miR-449b
BCL11B	3	miR-30b,miR-30a-3p,miR-429
BNC2	3	miR-500,miR-449b,miR-429
BRWD3	3	miR-30b,miR-30a-3p,miR-429
C11orf41	3	miR-221,miR-335,miR-483-3p
C15orf38-AP3S2	3	miR-541,miR-449b,miR-511
C1orf21	3	miR-335,miR-328,miR-888
C6orf168	3	miR-500,miR-545,miR-499-5p
C6orf89	3	miR-30b,miR-221,miR-30a-3p
CBL	3	miR-545,miR-511,miR-429
CDK19	3	miR-500,miR-668,miR-221
CELF2	3	miR-545,miR-511,miR-888
CPSF6	3	miR-545,miR-30b,miR-30a-3p
CREBZF	3	miR-545,miR-221,miR-499-5p
DSC3	3	miR-449b,miR-429,miR-888

ENAH	3	miR-545,miR-429,miR-888
ENTPD7	3	miR-545,miR-221,miR-511
EPM2AIP1	3	miR-500,miR-499-5p,miR-429
FAM123B	3	miR-328,miR-449b,miR-511
FAT3	3	miR-545,miR-449b,miR-429
FBXO32	3	miR-30b,miR-30a-3p,miR-511
GTPBP10	3	miR-500,miR-511,miR-429
HNRNPA3	3	miR-545,miR-30b,miR-30a-3p
HUNK	3	miR-500,miR-545,miR-335
IGF2BP1	3	miR-545,miR-541,miR-511
KCNN3	3	miR-668,miR-499-5p,miR-483-3p
KIAA0513	3	miR-668,miR-335,miR-328
KIAA1462	3	miR-335,miR-511,miR-429
KPNA4	3	miR-499-5p,miR-541,miR-888
LYRM2	3	miR-500,miR-499-5p,miR-429
MED12L	3	miR-545,miR-30b,miR-30a-3p
MMAA	3	miR-500,miR-335,miR-770-5p
MTX3	3	miR-545,miR-151-3p,miR-511
NAA38	3	miR-500,miR-545,miR-429
NUFIP2	3	miR-545,miR-221,miR-499-5p
PAG1	3	miR-668,miR-511,miR-888
PALM2	3	miR-30b,miR-30a-3p,miR-511
PAPOLG	3	miR-30b,miR-30a-3p,miR-151-3p
PEAK1	3	miR-545,miR-335,miR-449b
PEG10	3	miR-545,miR-30b,miR-30a-3p
PHACTR2	3	miR-770-5p,miR-511,miR-429
PHC3	3	miR-500,miR-499-5p,miR-429
PHF15	3	miR-545,miR-541,miR-449b
PLAGL2	3	miR-30b,miR-30a-3p,miR-541
PPARGC1B	3	miR-30b,miR-335,miR-30a-3p
PPM1A	3	miR-449b,miR-429,miR-888
PRLR	3	miR-545,miR-30b,miR-30a-3p
PRR11	3	miR-30b,miR-30a-3p,miR-449b
PRTG	3	miR-30b,miR-30a-3p,miR-429
PRUNE2	3	miR-30b,miR-335,miR-30a-3p
RAB22A	3	miR-500,miR-151-3p,miR-511
RAB27B	3	miR-500,miR-511,miR-888
RBM12	3	miR-500,miR-30b,miR-30a-3p
RIMS3	3	miR-545,miR-221,miR-449b
ROD1	3	miR-500,miR-545,miR-221
RORA	3	miR-30b,miR-30a-3p,miR-888

RUNX2	3	miR-30b,miR-30a-3p,miR-511
S100PBP	3	miR-30b,miR-30a-3p,miR-541
SECISBP2L	3	miR-335,miR-499-5p,miR-429
SLC22A23	3	miR-30b,miR-335,miR-30a-3p
SLC24A2	3	miR-541,miR-511,miR-888
SLC35B4	3	miR-30b,miR-30a-3p,miR-429
SLC6A6	3	miR-30b,miR-30a-3p,miR-511
SLC7A11	3	miR-30b,miR-30a-3p,miR-429
ST8SIA3	3	miR-499-5p,miR-511,miR-888
STXBP5	3	miR-545,miR-30b,miR-30a-3p
TC2N	3	miR-30b,miR-30a-3p,miR-429
TMEM237	3	miR-500,miR-545,miR-888
TRIM33	3	miR-545,miR-429,miR-888
TSC1	3	miR-221,miR-335,miR-151-3p
UBE2QL1	3	miR-500,miR-449b,miR-511
VGLL3	3	miR-500,miR-511,miR-888
XPO4	3	miR-545,miR-499-5p,miR-888
ZBTB41	3	miR-500,miR-30b,miR-30a-3p
ZC3H6	3	miR-545,miR-429,miR-888
ZNF761	3	miR-30b,miR-770-5p,miR-30a-3p
ZYG11B	3	miR-511,miR-429,miR-888
AADACL3	2	miR-545,miR-511
ADAMTS5	2	miR-511,miR-888
ADRBK2	2	miR-545,miR-888
AFF3	2	miR-30b,miR-30a-3p
AGFG1	2	miR-221,miR-429
AIDA	2	miR-30b,miR-30a-3p
ANKRA2	2	miR-30b,miR-30a-3p
ANKRD20A1	2	miR-30b,miR-30a-3p
ANKRD20A2	2	miR-30b,miR-30a-3p
ANKRD20A3	2	miR-30b,miR-30a-3p
ANKRD20A4	2	miR-30b,miR-30a-3p
ANKRD22	2	miR-30b,miR-30a-3p
ANKRD52	2	miR-541,miR-449b
ANTXR2	2	miR-770-5p,miR-499-5p
ARHGAP19	2	miR-545,miR-429
ASB1	2	miR-545,miR-449b
ATAD2B	2	miR-30b,miR-30a-3p
ATP7A	2	miR-499-5p,miR-888
ATRX	2	miR-511,miR-888
ATXN1	2	miR-221,miR-429

AVEN	2	miR-30b,miR-30a-3p
B3GNT5	2	miR-30b,miR-30a-3p
BACH2	2	miR-545,miR-511
BCAP29	2	miR-30b,miR-30a-3p
BNIP3L	2	miR-30b,miR-30a-3p
BRAP	2	miR-30b,miR-30a-3p
C11orf95	2	miR-449b,miR-429
C1orf183	2	miR-328,miR-449b
C20orf177	2	miR-545,miR-429
C5orf24	2	miR-511,miR-429
C6orf174	2	miR-511,miR-429
C7orf31	2	miR-30b,miR-30a-3p
CACNA1C	2	miR-545,miR-668
CALU	2	miR-335,miR-449b
CCDC117	2	miR-30b,miR-30a-3p
CCL22	2	miR-541,miR-449b
CCND1	2	miR-668,miR-511
CCNG2	2	miR-335,miR-770-5p
CD47	2	miR-668,miR-449b
CD80	2	miR-30b,miR-30a-3p
CD84	2	miR-511,miR-429
CELSR3	2	miR-30b,miR-30a-3p
CLDN1	2	miR-151-3p,miR-888
CLDN18	2	miR-449b,miR-888
CNKSR2	2	miR-30b,miR-30a-3p
CNOT6L	2	miR-429,miR-888
CNR1	2	miR-30b,miR-30a-3p
COL4A4	2	miR-449b,miR-511
CXorf23	2	miR-499-5p,miR-511
CYP24A1	2	miR-30b,miR-30a-3p
DBT	2	miR-221,miR-151-3p
DIEXF	2	miR-500,miR-449b
E2F7	2	miR-30b,miR-30a-3p
EEA1	2	miR-30b,miR-30a-3p
ELK4	2	miR-335,miR-429
ELL2	2	miR-30b,miR-30a-3p
ELMOD2	2	miR-511,miR-429
EMCN	2	miR-30b,miR-30a-3p
ENTPD1	2	miR-335,miR-511
EPHA7	2	miR-511,miR-888
ERG	2	miR-499-5p,miR-511

FAHD1	2	miR-30b,miR-30a-3p
FAM126B	2	miR-429,miR-888
FAM133A	2	miR-30b,miR-30a-3p
FAM168B	2	miR-328,miR-888
FAM190B	2	miR-545,miR-511
FAM81A	2	miR-30b,miR-30a-3p
FBXW2	2	miR-500,miR-335
FGD4	2	miR-545,miR-499-5p
FGF2	2	miR-545,miR-499-5p
FGF9	2	miR-499-5p,miR-511
FLRT2	2	miR-545,miR-511
FOXN2	2	miR-499-5p,miR-888
FRS2	2	miR-221,miR-429
FZR1	2	miR-541,miR-449b
GABRG1	2	miR-500,miR-888
GAS7	2	miR-541,miR-888
GCET2	2	miR-30b,miR-30a-3p
GDAP1	2	miR-499-5p,miR-888
GGCX	2	miR-221,miR-541
GJA3	2	miR-429,miR-888
GLDN	2	miR-545,miR-888
GMFB	2	miR-770-5p,miR-429
GNA13	2	miR-30b,miR-30a-3p
GNL3L	2	miR-335,miR-888
GOLGA6C	2	miR-429,miR-888
GTF2H5	2	miR-545,miR-511
GXYLT1	2	miR-429,miR-888
H6PD	2	miR-545,miR-449b
HTR1F	2	miR-30b,miR-30a-3p
IL6R	2	miR-335,miR-541
IPMK	2	miR-511,miR-429
IRF4	2	miR-545,miR-449b
ISY1-RAB43	2	miR-541,miR-449b
ITGA2	2	miR-30b,miR-30a-3p
ITPK1	2	miR-30b,miR-30a-3p
IYD	2	miR-668,miR-429
JHDM1D	2	miR-499-5p,miR-429
KCNQ3	2	miR-221,miR-888
KCTD12	2	miR-429,miR-888
KIAA0319	2	miR-30b,miR-30a-3p
KIAA1549	2	miR-545,miR-499-5p

KIAA1949	2	miR-30b,miR-30a-3p
KIF17	2	miR-541,miR-449b
KIF1B	2	miR-545,miR-335
KIF3A	2	miR-335,miR-888
KLHL25	2	miR-541,miR-449b
KLHL28	2	miR-30b,miR-30a-3p
LIN28A	2	miR-30b,miR-30a-3p
LOC100132146	2	miR-30b,miR-30a-3p
MAP3K2	2	miR-30b,miR-30a-3p
MAP4K5	2	miR-429,miR-888
MAP9	2	miR-30b,miR-30a-3p
MBNL3	2	miR-511,miR-429
MIER3	2	miR-30b,miR-30a-3p
MKI67	2	miR-545,miR-668
MKL2	2	miR-545,miR-511
MKRN3	2	miR-30b,miR-30a-3p
MLEC	2	miR-541,miR-888
MMAB	2	miR-335,miR-449b
MTDH	2	miR-30b,miR-30a-3p
MTMR9	2	miR-545,miR-429
MXD4	2	miR-541,miR-449b
N6AMT1	2	miR-30b,miR-30a-3p
NAT8L	2	miR-541,miR-449b
NECAB1	2	miR-668,miR-429
NEDD4	2	miR-30b,miR-30a-3p
NOS1AP	2	miR-545,miR-449b
ORAI2	2	miR-335,miR-541
OSGIN2	2	miR-30b,miR-30a-3p
PAFAH1B2	2	miR-30b,miR-30a-3p
PAPD5	2	miR-429,miR-888
PCGF5	2	miR-335,miR-888
PCMTD2	2	miR-30b,miR-30a-3p
PDE4D	2	miR-30b,miR-30a-3p
PGR	2	miR-511,miR-888
PHIP	2	miR-30b,miR-30a-3p
PHTF2	2	miR-30b,miR-30a-3p
PI15	2	miR-500,miR-499-5p
PIP4K2A	2	miR-30b,miR-30a-3p
PLEKHA6	2	miR-221,miR-541
PLXDC1	2	miR-545,miR-511
PPARA	2	miR-500,miR-449b

PPARGC1A	2	miR-30b,miR-30a-3p
PPIE	2	miR-545,miR-449b
PPP1R9A	2	miR-429,miR-888
PRDM1	2	miR-30b,miR-30a-3p
PRPF4B	2	miR-545,miR-511
PTGFRN	2	miR-30b,miR-30a-3p
PTPN14	2	miR-511,miR-429
PVRL1	2	miR-541,miR-449b
QKI	2	miR-511,miR-429
RAB11FIP2	2	miR-545,miR-429
RAB43	2	miR-541,miR-449b
RALGAPB	2	miR-668,miR-335
RBAK	2	miR-500,miR-888
REEP3	2	miR-30b,miR-30a-3p
RIMBP2	2	miR-30b,miR-30a-3p
RNF165	2	miR-30b,miR-30a-3p
SAMD4A	2	miR-500,miR-888
SBNO1	2	miR-545,miR-888
SC5DL	2	miR-545,miR-511
SCAI	2	miR-545,miR-429
SCML2	2	miR-30b,miR-30a-3p
SCN2A	2	miR-30b,miR-30a-3p
SCN2B	2	miR-541,miR-449b
SEMA3D	2	miR-511,miR-888
SEMA4F	2	miR-449b,miR-888
SERTAD2	2	miR-511,miR-888
SHISA7	2	miR-541,miR-449b
SHROOM4	2	miR-151-3p,miR-429
SIKE1	2	miR-499-5p,miR-429
SLC2A13	2	miR-221,miR-151-3p
SLC36A1	2	miR-30b,miR-30a-3p
SLC4A4	2	miR-429,miR-888
SLC4A7	2	miR-221,miR-429
SMCR7L	2	miR-545,miR-429
SNTB2	2	miR-429,miR-888
SNX16	2	miR-30b,miR-30a-3p
SOS1	2	miR-151-3p,miR-429
SPRED1	2	miR-770-5p,miR-888
ST3GAL1	2	miR-668,miR-449b
ST8SIA1	2	miR-30b,miR-30a-3p
STIM2	2	miR-30b,miR-30a-3p

STS	2	miR-30b,miR-30a-3p
STX16	2	miR-30b,miR-30a-3p
SV2B	2	miR-500,miR-888
TEAD1	2	miR-500,miR-335
TET2	2	miR-545,miR-511
TFCP2L1	2	miR-541,miR-449b
THRB	2	miR-511,miR-429
THSD7A	2	miR-545,miR-429
TMEM181	2	miR-30b,miR-30a-3p
TMEM194A	2	miR-545,miR-335
TMEM229A	2	miR-30b,miR-30a-3p
TMEM56	2	miR-30b,miR-30a-3p
TMEM87B	2	miR-30b,miR-30a-3p
TMTC3	2	miR-668,miR-888
TNFSF13B	2	miR-30b,miR-30a-3p
TP53INP1	2	miR-30b,miR-30a-3p
TPCN2	2	miR-483-3p,miR-449b
TRAM2	2	miR-500,miR-888
TRIM67	2	miR-335,miR-541
TWF1	2	miR-30b,miR-30a-3p
USP37	2	miR-30b,miR-30a-3p
USP45	2	miR-30b,miR-30a-3p
VANGL1	2	miR-545,miR-511
VAPA	2	miR-335,miR-499-5p
VAPB	2	miR-221,miR-483-3p
VPS37C	2	miR-328,miR-888
WDFY3	2	miR-30b,miR-30a-3p
WDR7	2	miR-30b,miR-30a-3p
WNK3	2	miR-545,miR-429
XYLT1	2	miR-449b,miR-511
YAF2	2	miR-30b,miR-30a-3p
YPEL2	2	miR-30b,miR-30a-3p
ZDHHC20	2	miR-30b,miR-30a-3p
ZNF117	2	miR-770-5p,miR-511
ZNF275	2	miR-541,miR-449b
ZNF480	2	miR-770-5p,miR-511
ZNF516	2	miR-668,miR-429
ZNF518A	2	miR-30b,miR-30a-3p
ZNF621	2	miR-770-5p,miR-429
ZNF704	2	miR-221,miR-511
ZNF765	2	miR-770-5p,miR-511

ABCC5	1	miR-545
ABL1	1	miR-335
ABO	1	miR-511
ACADSB	1	miR-511
ACOT13	1	miR-511
ACPL2	1	miR-545
ACSS1	1	miR-335
ACTC1	1	miR-429
ADAM19	1	miR-545
ADAM33	1	miR-511
ADAT2	1	miR-545
AFF4	1	miR-151-3p
AK4	1	miR-668
ALCAM	1	miR-511
ALDH5A1	1	miR-888
ALDH7A1	1	miR-335
ALS2CR8	1	miR-429
ANK3	1	miR-449b
ANKRD40	1	miR-499-5p
ANKS1A	1	miR-449b
APOOL	1	miR-429
AQP4	1	miR-511
AQPEP	1	miR-511
ARHGAP26	1	miR-545
ARHGAP29	1	miR-335
ARHGAP42	1	miR-545
ARHGEF12	1	miR-888
ARHGEF6	1	miR-545
ARID5B	1	miR-888
ARIH1	1	miR-545
ARIH2	1	miR-429
ARL11	1	miR-511
ARL3	1	miR-545
ARL4A	1	miR-545
ARL5A	1	miR-429
ARNT	1	miR-545
ARRB1	1	miR-545
ARRDC3	1	miR-545
ARSB	1	miR-449b
ATF7	1	miR-449b
ATP11A	1	miR-545

ATP11B	1	miR-429
ATP2C1	1	miR-888
ATP9A	1	miR-335
ATRNL1	1	miR-511
ATXN1L	1	miR-545
ATXN7L1	1	miR-545
ATXN7L3	1	miR-449b
B3GALTL	1	miR-429
B4GALT6	1	miR-429
BANP	1	miR-545
BCL2L11	1	miR-429
BCL2L15	1	miR-545
BDP1	1	miR-429
BET1L	1	miR-545
BMPR1B	1	miR-511
BMPR2	1	miR-545
BMS1	1	miR-668
BVES	1	miR-545
C10orf47	1	miR-499-5p
C12orf51	1	miR-888
C13orf31	1	miR-888
C14orf101	1	miR-511
C14orf147	1	miR-335
C14orf43	1	miR-545
C16orf52	1	miR-429
C16orf58	1	miR-449b
C16orf72	1	miR-429
C17orf75	1	miR-499-5p
C18orf1	1	miR-545
C18orf42	1	miR-668
C19orf12	1	miR-429
C1QTNF7	1	miR-511
C1orf115	1	miR-545
C1orf116	1	miR-335
C1orf96	1	miR-429
C21orf91	1	miR-429
C2orf69	1	miR-511
C3orf33	1	miR-335
C3orf64	1	miR-221
C6	1	miR-221
C6orf120	1	miR-429

C6orf35	1	miR-545
C7orf58	1	miR-429
C9orf5	1	miR-888
C9orf66	1	miR-511
CABP4	1	miR-511
CADM1	1	miR-545
CALCRL	1	miR-545
CAMK2D	1	miR-545
CAMSAP1L1	1	miR-429
CARKD	1	miR-511
CASC4	1	miR-429
CASK	1	miR-499-5p
CCDC102B	1	miR-511
CCDC113	1	miR-499-5p
CCDC144A	1	miR-545
CCDC47	1	miR-888
CCDC85C	1	miR-335
CCNJ	1	miR-429
CCNT2	1	miR-335
CCP110	1	miR-511
CCPG1	1	miR-545
CCR5	1	miR-511
CD209	1	miR-335
CD28	1	miR-888
CDC42SE2	1	miR-545
CDC73	1	miR-429
CDH6	1	miR-429
CERS3	1	miR-545
CFL2	1	miR-429
CHFR	1	miR-668
CHM	1	miR-429
CHN2	1	miR-545
CHST11	1	miR-449b
CHST6	1	miR-511
CLCC1	1	miR-429
CLCN5	1	miR-151-3p
CLEC5A	1	miR-888
CLIC5	1	miR-511
CMTM4	1	miR-545
COL19A1	1	miR-511
COL4A3BP	1	miR-429

COX11	1	miR-429
CPLX2	1	miR-449b
CPM	1	miR-499-5p
CPPED1	1	miR-545
CRY2	1	miR-449b
CSDC2	1	miR-449b
CSMD2	1	miR-511
CSNK1G3	1	miR-545
CTIF	1	miR-449b
CYLD	1	miR-335
CYP1B1	1	miR-429
CYP20A1	1	miR-888
DAZ1	1	miR-429
DAZ2	1	miR-429
DAZ3	1	miR-429
DAZ4	1	miR-429
DCBLD2	1	miR-429
DCC	1	miR-888
DCTN4	1	miR-335
DCX	1	miR-429
DERL1	1	miR-888
DGCR14	1	miR-545
DGKE	1	miR-429
DIRAS2	1	miR-429
DIS3	1	miR-668
DLG2	1	miR-511
DLL1	1	miR-449b
DNA2	1	miR-429
DNAJC11	1	miR-545
DNAJC3	1	miR-429
DNM3	1	miR-545
DOK6	1	miR-545
DPH3	1	miR-545
DPP10	1	miR-888
DPY19L1	1	miR-545
DRP2	1	miR-429
DUSP4	1	miR-511
DUSP8	1	miR-449b
DZIP1	1	miR-429
EDA	1	miR-545
EDNRA	1	miR-429

EEF1A1	1	miR-151-3p
EFNB1	1	miR-449b
EIF2A	1	miR-511
EIF2C4	1	miR-545
EIF2S3	1	miR-221
EIF4E	1	miR-545
EIF4E3	1	miR-545
ELAVL3	1	miR-511
ELF4	1	miR-545
EML6	1	miR-221
ENPP1	1	miR-545
EPT1	1	miR-335
ERCC4	1	miR-499-5p
ERI1	1	miR-429
ERLIN2	1	miR-499-5p
ERMN	1	miR-888
ERRFI1	1	miR-429
ESRP2	1	miR-511
ESRRG	1	miR-429
ESYT3	1	miR-449b
ETNK1	1	miR-511
ETV1	1	miR-545
FADS1	1	miR-511
FAM105B	1	miR-429
FAM107A	1	miR-449b
FAM110C	1	miR-545
FAM115A	1	miR-511
FAM129A	1	miR-511
FAM131B	1	miR-449b
FAM13A	1	miR-545
FAM167A	1	miR-449b
FAM169A	1	miR-429
FAM22D	1	miR-449b
FAM46A	1	miR-221
FAM46C	1	miR-888
FAM55C	1	miR-335
FAM59A	1	miR-151-3p
FAM71D	1	miR-511
FAM76A	1	miR-449b
FAM8A1	1	miR-429
FASTKD2	1	miR-511

FBXO9	1	miR-888
FEM1C	1	miR-151-3p
FGD3	1	miR-449b
FGD6	1	miR-429
FGF12	1	miR-511
FGFR1	1	miR-545
FKTN	1	miR-888
FLOT2	1	miR-449b
FMN1	1	miR-335
FMNL3	1	miR-449b
FNBP1L	1	miR-511
FNDC3B	1	miR-429
FOXP2	1	miR-511
FRAS1	1	miR-429
FREM2	1	miR-335
FSD1L	1	miR-429
FUT4	1	miR-429
G3BP2	1	miR-888
GAB1	1	miR-429
GABRA1	1	miR-221
GABRB3	1	miR-511
GAS8	1	miR-449b
GATC	1	miR-449b
GBP4	1	miR-545
GDAP2	1	miR-888
GFPT1	1	miR-429
GLG1	1	miR-449b
GLIS3	1	miR-511
GLO1	1	miR-335
GLYCTK	1	miR-335
GNAI3	1	miR-545
GNE	1	miR-888
GNL1	1	miR-545
GNRHR	1	miR-449b
GNS	1	miR-335
GOLGA1	1	miR-221
GOPC	1	miR-511
GPC6	1	miR-429
GPD1	1	miR-545
GPD2	1	miR-545
GPM6A	1	miR-429

GPR107	1	miR-429
GRB2	1	miR-545
GRIK3	1	miR-545
GRSF1	1	miR-545
HAUS2	1	miR-545
HAUS3	1	miR-335
HBS1L	1	miR-429
HCN3	1	miR-449b
HDAC2	1	miR-511
HDX	1	miR-511
HEG1	1	miR-429
HEMK1	1	miR-335
HIGD1A	1	miR-545
HLF	1	miR-335
HMGB1	1	miR-545
HRH1	1	miR-511
ICA1L	1	miR-429
IDH3A	1	miR-511
IFNAR1	1	miR-545
IFRD1	1	miR-221
IGDCC4	1	miR-511
IKZF1	1	miR-888
IKZF2	1	miR-499-5p
IL10RA	1	miR-545
IL17RD	1	miR-335
IL2RB	1	miR-449b
IL4R	1	miR-545
ILDR2	1	miR-545
IMPG2	1	miR-499-5p
IPO7	1	miR-429
IPPK	1	miR-545
IRAK3	1	miR-449b
IRF2BP2	1	miR-511
ITGAV	1	miR-429
ITGB1	1	miR-888
ITGB1BP1	1	miR-668
ITGB8	1	miR-499-5p
JPH1	1	miR-545
KATNAL1	1	miR-429
KBTBD6	1	miR-429
KCNA1	1	miR-511

KCNH1	1	miR-221
KCNK3	1	miR-449b
KCNMA1	1	miR-511
KCTD20	1	miR-888
KCTD7	1	miR-335
KCTD8	1	miR-429
KDM5A	1	miR-888
KHDRBS2	1	miR-511
KIAA0408	1	miR-429
KIAA0415	1	miR-668
KIAA1147	1	miR-511
KIAA1430	1	miR-429
KIAA1432	1	miR-429
KIAA1737	1	miR-545
KIAA2022	1	miR-545
KLHL18	1	miR-335
KLHL24	1	miR-511
KLHL4	1	miR-511
KLHL5	1	miR-511
KPNA6	1	miR-335
KRT77	1	miR-545
KY	1	miR-449b
L2HGDH	1	miR-888
LANCL2	1	miR-511
LDB2	1	miR-511
LHFPL2	1	miR-888
LIFR	1	miR-668
LIMA1	1	miR-499-5p
LIN7C	1	miR-499-5p
LMO3	1	miR-511
LMX1A	1	miR-335
LOC100131094	1	miR-449b
LOC388630	1	miR-545
LOC389831	1	miR-511
LOX	1	miR-429
LRP6	1	miR-545
LRP8	1	miR-888
LRRC14	1	miR-449b
LRRC55	1	miR-449b
LRRC58	1	miR-429
LRRC8C	1	miR-888

LRRCC1	1	miR-221
LSAMP	1	miR-511
LYPD6	1	miR-511
MAP2	1	miR-429
MAP4K4	1	miR-511
MAPK14	1	miR-545
MAPK1IP1L	1	miR-511
MAPT	1	miR-449b
MARK1	1	miR-221
MBL2	1	miR-499-5p
MBLAC2	1	miR-429
MBOAT2	1	miR-545
MCFD2	1	miR-429
MDM2	1	miR-511
MED13	1	miR-429
METTL16	1	miR-545
METTL7A	1	miR-335
MFAP5	1	miR-545
MIB1	1	miR-888
MIPOL1	1	miR-429
MKLN1	1	miR-429
MMP16	1	miR-511
MMS22L	1	miR-429
MPP2	1	miR-449b
MRE11A	1	miR-335
MRO	1	miR-545
MSN	1	miR-429
MTMR3	1	miR-668
MUTED	1	miR-545
MXI1	1	miR-511
MYBL1	1	miR-221
MYEF2	1	miR-499-5p
MYLK	1	miR-545
MYNN	1	miR-888
MYO5B	1	miR-545
MYOCD	1	miR-511
NANOS1	1	miR-429
NAP1L1	1	miR-511
NAV1	1	miR-449b
NAV3	1	miR-449b
NCALD	1	miR-511

NCOA3	1	miR-429
NCOA7	1	miR-499-5p
NDST1	1	miR-449b
NEBL	1	miR-335
NEDD4L	1	miR-545
NEURL1B	1	miR-449b
NEUROD2	1	miR-511
NF1	1	miR-429
NFATC2IP	1	miR-668
NFYB	1	miR-888
NICN1	1	miR-335
NIPA1	1	miR-335
NLGN4X	1	miR-429
NR1D2	1	miR-511
NR2C2	1	miR-429
NR5A2	1	miR-429
NRAS	1	miR-888
NRXN3	1	miR-511
NUCKS1	1	miR-335
ODZ4	1	miR-668
OMG	1	miR-499-5p
OPCML	1	miR-511
OPN5	1	miR-511
ORC4	1	miR-511
OSBPL3	1	miR-221
OSTM1	1	miR-429
OTUD1	1	miR-545
OTUD7B	1	miR-429
OXTR	1	miR-511
PAIP2B	1	miR-499-5p
PAK2	1	miR-888
PARD3	1	miR-511
PARP11	1	miR-511
PAX8	1	miR-511
PCDH19	1	miR-545
PCDHB9	1	miR-511
PCGF3	1	miR-545
PCNX	1	miR-429
PCP4L1	1	miR-151-3p
PDCD4	1	miR-499-5p
PDE10A	1	miR-545

PDE11A	1	miR-511
PDE5A	1	miR-429
PDP2	1	miR-668
PDXDC1	1	miR-545
PELI2	1	miR-429
PER2	1	miR-449b
PERP	1	miR-888
PEX19	1	miR-888
PEX26	1	miR-545
PGM2L1	1	miR-545
PGM3	1	miR-151-3p
PHLDA1	1	miR-545
PIGM	1	miR-429
PIK3AP1	1	miR-545
PKD2	1	miR-888
PKHD1	1	miR-545
PKNOX1	1	miR-449b
PLA2G12A	1	miR-545
PLA2G2F	1	miR-449b
PLAG1	1	miR-545
PLCXD3	1	miR-221
PLEKHB2	1	miR-545
PLEKHM3	1	miR-511
PM20D2	1	miR-511
PMPCB	1	miR-429
POLK	1	miR-429
POTEM	1	miR-545
PPHLN1	1	miR-151-3p
PPP1CB	1	miR-429
PPP2R1B	1	miR-429
PRDM16	1	miR-511
PRDM2	1	miR-335
PRDM7	1	miR-511
PRKCB	1	miR-511
PRKX	1	miR-545
PRR14L	1	miR-499-5p
PRR5L	1	miR-335
PSD3	1	miR-668
PSEN1	1	miR-545
PTCH1	1	miR-545
PTPN18	1	miR-449b

PTPRB	1	miR-335
PYROXD1	1	miR-511
ProSAPiP1	1	miR-449b
RAB10	1	miR-545
RAB11A	1	miR-888
RAB2A	1	miR-511
RAB33B	1	miR-429
RAB9B	1	miR-668
RAPGEF2	1	miR-511
RASGRP3	1	miR-888
RASSF6	1	miR-511
RBBP4	1	miR-429
RBBP9	1	miR-545
RBM15B	1	miR-511
RBM20	1	miR-545
RBM8A	1	miR-429
RBMS2	1	miR-545
RCN2	1	miR-888
RECK	1	miR-429
REEP1	1	miR-545
RFX7	1	miR-511
RGAG1	1	miR-335
RGS6	1	miR-221
RHBDD1	1	miR-499-5p
RHOU	1	miR-335
RIF1	1	miR-545
RIMS1	1	miR-499-5p
RNF169	1	miR-499-5p
RNF182	1	miR-888
RNF19A	1	miR-429
RNF2	1	miR-429
RNF38	1	miR-545
ROBO2	1	miR-888
ROCK2	1	miR-429
RPS23	1	miR-429
RPS6KA2	1	miR-545
RPS6KA3	1	miR-429
RPS6KB1	1	miR-429
RPS6KL1	1	miR-511
RUNX1T1	1	miR-221
RYBP	1	miR-511

SAMD8	1	miR-888
SAR1B	1	miR-888
SASH1	1	miR-511
SATB2	1	miR-511
SCAMP1	1	miR-429
SCNN1G	1	miR-449b
SDK2	1	miR-449b
SEC14L5	1	miR-335
SEC61A1	1	miR-449b
SEC62	1	miR-221
SEL1L	1	miR-668
SEMA3A	1	miR-499-5p
SEMA5A	1	miR-429
SEPT2	1	miR-888
SEPT3	1	miR-449b
SESTD1	1	miR-449b
SGCD	1	miR-511
SH2D1A	1	miR-511
SHC3	1	miR-511
SHC4	1	miR-429
SKA3	1	miR-429
SKAP2	1	miR-668
SKP1	1	miR-499-5p
SLAIN2	1	miR-511
SLC12A2	1	miR-888
SLC12A6	1	miR-888
SLC15A5	1	miR-429
SLC1A4	1	miR-545
SLC25A35	1	miR-335
SLC26A2	1	miR-151-3p
SLC30A4	1	miR-499-5p
SLC35E1	1	miR-545
SLC35E2	1	miR-545
SLC35F5	1	miR-429
SLC41A2	1	miR-545
SLC5A1	1	miR-545
SLC5A12	1	miR-511
SLC6A3	1	miR-449b
SLC7A14	1	miR-511
SLC8A1	1	miR-151-3p
SLC9A8	1	miR-545

SLCO2A1	1	miR-449b
SMAD2	1	miR-511
SMAD5	1	miR-429
SMARCA5	1	miR-511
SMU1	1	miR-545
SMURF1	1	miR-545
SNAP25	1	miR-545
SNAP29	1	miR-545
SNRK	1	miR-499-5p
SNX22	1	miR-511
SOCS4	1	miR-429
SOCS5	1	miR-888
SOCS7	1	miR-449b
SOX6	1	miR-499-5p
SPATA5	1	miR-511
SPDYE5	1	miR-545
SPOPL	1	miR-511
SRC	1	miR-449b
ST18	1	miR-511
STAC2	1	miR-545
STAM2	1	miR-429
STARD3	1	miR-449b
STK38L	1	miR-888
STK4	1	miR-335
STRBP	1	miR-545
STX11	1	miR-449b
STX7	1	miR-545
STXBP4	1	miR-151-3p
STYX	1	miR-429
SUMO3	1	miR-545
SUN2	1	miR-335
SUPT7L	1	miR-499-5p
SUSD5	1	miR-429
SYN2	1	miR-335
SYNJ1	1	miR-545
SYT1	1	miR-449b
SYT2	1	miR-545
SYT4	1	miR-888
TAF9B	1	miR-545
TBC1D24	1	miR-888
TBC1D8B	1	miR-545

TBX18	1	miR-429
TCEANC2	1	miR-545
TCF4	1	miR-511
TERF1	1	miR-429
TFAP2A	1	miR-429
TFDP2	1	miR-335
TFEC	1	miR-429
TFPI	1	miR-545
TGFB3	1	miR-511
TGFBR1	1	miR-545
TGIF2	1	miR-545
THAP2	1	miR-429
THBS2	1	miR-545
THSD4	1	miR-449b
TK2	1	miR-668
TLCD2	1	miR-511
TLL2	1	miR-429
TLR4	1	miR-511
TMCC1	1	miR-888
TMED5	1	miR-511
TMED7	1	miR-429
TMEM110	1	miR-545
TMEM130	1	miR-449b
TMEM140	1	miR-511
TMEM167A	1	miR-429
TMEM184B	1	miR-449b
TMEM213	1	miR-335
TMEM215	1	miR-545
TMEM26	1	miR-429
TMEM41A	1	miR-335
TMEM47	1	miR-499-5p
TMOD3	1	miR-429
TMX1	1	miR-429
TNFAIP1	1	miR-335
TNKS	1	miR-449b
TNPO1	1	miR-151-3p
TNRC6C	1	miR-511
TOB2	1	miR-449b
TP53BP2	1	miR-221
TPM3	1	miR-888
TPPP	1	miR-449b

TRERF1	1	miR-545
TRHDE	1	miR-888
TRMT5	1	miR-429
TRPM7	1	miR-888
TSGA14	1	miR-429
TTC39C	1	miR-545
U2SURP	1	miR-511
UBE2H	1	miR-335
UBFD1	1	miR-888
UCN2	1	miR-449b
UGGT1	1	miR-668
ULK2	1	miR-335
UNC5D	1	miR-335
UNC80	1	miR-545
USP13	1	miR-335
USP15	1	miR-545
USP51	1	miR-511
USP9X	1	miR-545
UTY	1	miR-429
VAMP2	1	miR-449b
VASH2	1	miR-429
VAV3	1	miR-499-5p
VMA21	1	miR-888
VPS37B	1	miR-449b
VPS4A	1	miR-449b
VTI1A	1	miR-429
WAPAL	1	miR-429
WASF3	1	miR-429
WDFY1	1	miR-888
WDR72	1	miR-888
WDR82	1	miR-429
WEE1	1	miR-545
WIPF1	1	miR-429
WWC2	1	miR-499-5p
WWC3	1	miR-449b
XIRP1	1	miR-449b
XKR9	1	miR-888
XPR1	1	miR-511
XRCC2	1	miR-511
XRN1	1	miR-888
YPEL1	1	miR-668

ZBTB24	1	miR-511
ZBTB34	1	miR-668
ZBTB38	1	miR-888
ZBTB46	1	miR-545
ZBTB8A	1	miR-511
ZC3HAV1	1	miR-545
ZDHHC17	1	miR-429
ZDHHC23	1	miR-888
ZEB1	1	miR-429
ZEB2	1	miR-429
ZFAND5	1	miR-221
ZFPM2	1	miR-429
ZFX	1	miR-429
ZHX2	1	miR-449b
ZIC1	1	miR-545
ZNF12	1	miR-668
ZNF148	1	miR-545
ZNF217	1	miR-499-5p
ZNF24	1	miR-545
ZNF280C	1	miR-511
ZNF470	1	miR-888
ZNF512B	1	miR-449b
ZNF532	1	miR-429
ZNF562	1	miR-511
ZNF641	1	miR-888
ZNF662	1	miR-545
ZNF670	1	miR-499-5p
ZNF678	1	miR-429
ZNF681	1	miR-545
ZNF697	1	miR-429
ZNF737	1	miR-335
ZNF793	1	miR-888
ZNF814	1	miR-221
ZNF831	1	miR-449b
ZNRF1	1	miR-545
ZXDB	1	miR-221

Supplementary Table S5

Pathway	Percentage	num overlap	num in pathway	num miRNA targets	p value
KEGG_GLIOMA	0.41	26	64	2571	1.48E-07
KEGG_MELANOMA	0.39	26	67	2571	4.34E-07
KEGG_MAPK_SIGNALING_PATHWAY	0.25	66	261	2571	8.00E-07
KEGG_CHRONIC_MYELOID_LEUKEMIA	0.36	26	72	2571	2.18E-06
KEGG_INSULIN_SIGNALING_PATHWAY	0.29	39	136	2571	6.11E-06
KEGG_FOCAL_ADHESION	0.26	51	197	2571	6.74E-06
KEGG_PATHWAYS_IN_CANCER	0.23	74	321	2571	7.26E-06
KEGG_ERBB_SIGNALING_PATHWAY	0.33	28	86	2571	9.14E-06
KEGG_REGULATION_OF_ACTIN_CYTOSKELETON	0.25	52	206	2571	1.19E-05
KEGG_NEUROTROPHIN_SIGNALING_PATHWAY	0.29	36	125	2571	1.23E-05
KEGG_LONG_TERM_POTENTIATION	0.34	24	70	2571	1.47E-05
KEGG_AXON_GUIDANCE	0.28	36	129	2571	2.63E-05
KEGG_MTOR_SIGNALING_PATHWAY	0.37	19	51	2571	3.01E-05
KEGG_PHOSPHATIDYLINOSITOL_SIGNALING_SYSTEM	0.32	24	75	2571	5.34E-05
KEGG_PROSTATE_CANCER	0.29	25	87	2571	2.60E-04
KEGG_LONG_TERM_DEPRESSION	0.3	20	66	2571	4.89E-04
KEGG_FC_EPSILON_RI_SIGNALING_PATHWAY	0.29	22	76	2571	5.31E-04
KEGG_NON_SMALL_CELL_LUNG_CANCER	0.32	17	53	2571	6.16E-04
KEGG_GNRH_SIGNALING_PATHWAY	0.26	26	99	2571	9.04E-04
KEGG_PROGESTERONE_MEDIATED_OOCYTE_MATURATIO	0.27	22	82	2571	1.61E-03
KEGG_RENAL_CELL_CARCINOMA	0.28	19	69	2571	2.36E-03
KEGG_FC_GAMMA_R_MEDIATED_PHAGOCYTOSIS	0.25	24	95	2571	2.46E-03
KEGG_ENDOCYTOSIS	0.22	39	179	2571	2.90E-03
KEGG_ALDOSTERONE_REGULATED_SODIUM_REABSORPTI	0.32	13	41	2571	2.94E-03
KEGG_T_CELL_RECEPTOR_SIGNALING_PATHWAY	0.24	26	107	2571	2.99E-03
KEGG_SMALL_CELL_LUNG_CANCER	0.25	21	83	2571	4.36E-03
KEGG_HYPERTROPHIC_CARDIOMYOPATHY_HCM	0.25	21	83	2571	4.36E-03
KEGG_CELL_ADHESION_MOLECULES_CAMS	0.23	30	132	2571	4.40E-03
KEGG_B_CELL_RECEPTOR_SIGNALING_PATHWAY	0.26	19	74	2571	5.48E-03
KEGG_TGF_BETA_SIGNALING_PATHWAY	0.25	21	85	2571	5.85E-03
KEGG_GLYCEROPHOSPHOLIPID_METABOLISM	0.25	19	75	2571	6.40E-03
KEGG_VASCULAR_SMOOTH_MUSCLE_CONTRACTION	0.23	26	113	2571	6.47E-03
KEGG_GAP_JUNCTION	0.24	21	86	2571	6.74E-03
KEGG_COLORECTAL_CANCER	0.26	16	61	2571	8.33E-03

KEGG_ACUTE_MYELOID_LEUKEMIA	0.27	15	56	2571	8.53E-03
KEGG_ENDOMETRIAL_CANCER	0.27	14	51	2571	8.66E-03

Supplementary Table S6

Pathway	Percentage	num overlap	num in pathway	num miRNA targets	p value
KEGG_ENDOCYTOSIS	0.18	33	179	1589	2.50E-05
KEGG_ERBB_SIGNALING_PATHWAY	0.22	19	86	1589	1.12E-04
KEGG_MAPK_SIGNALING_PATHWAY	0.16	41	261	1589	1.34E-04
KEGG_AXON_GUIDANCE	0.19	24	129	1589	2.64E-04
KEGG_GNRH_SIGNALING_PATHWAY	0.2	20	99	1589	2.72E-04
KEGG_NEUROTROPHIN_SIGNALING_PATHWAY	0.18	23	125	1589	4.13E-04
KEGG_LONG_TERM_POTENTIATION	0.21	15	70	1589	8.04E-04
KEGG_GLIOMA	0.22	14	64	1589	9.59E-04
KEGG_CHRONIC_MYELOID_LEUKEMIA	0.21	15	72	1589	1.09E-03
KEGG_TGF_BETA_SIGNALING_PATHWAY	0.19	16	85	1589	2.33E-03
KEGG_SNARE_INTERACTIONS_IN_VESICULAR_TRANSPORT	0.25	9	36	1589	2.91E-03
KEGG_VASCULAR_SMOOTH_MUSCLE_CONTRACTION	0.17	19	113	1589	3.69E-03
KEGG_INSULIN_SIGNALING_PATHWAY	0.15	21	136	1589	6.52E-03

Supplementary Table S7

Pathway	Percentage	num overlap	num in pathway	num miRNA targets	p value
BIOCARTA_EIF4_PATHWAY	0.5	12	24	2571	2.89E-05
BIOCARTA_IL2RB_PATHWAY	0.39	15	38	2571	9.48E-05
BIOCARTA_CREB_PATHWAY	0.44	12	27	2571	1.23E-04
BIOCARTA_PTEN_PATHWAY	0.44	8	18	2571	1.72E-03
BIOCARTA_SPRY_PATHWAY	0.44	8	18	2571	1.72E-03
BIOCARTA_EDG1_PATHWAY	0.38	10	26	2571	1.76E-03
BIOCARTA_SRCRPTP_PATHWAY	0.55	6	11	2571	1.83E-03
BIOCARTA_AGR_PATHWAY	0.34	12	35	2571	2.01E-03
BIOCARTA_MYOSIN_PATHWAY	0.35	11	31	2571	2.24E-03
BIOCARTA_CTCF_PATHWAY	0.39	9	23	2571	2.59E-03
BIOCARTA_GH_PATHWAY	0.36	10	28	2571	3.34E-03
BIOCARTA_PYK2_PATHWAY	0.36	10	28	2571	3.34E-03
BIOCARTA_MET_PATHWAY	0.32	12	37	2571	3.41E-03
BIOCARTA_IGF1MTOR_PATHWAY	0.4	8	20	2571	3.81E-03
BIOCARTA_CBL_PATHWAY	0.46	6	13	2571	5.30E-03
BIOCARTA_ARENRF2_PATHWAY	0.46	6	13	2571	5.30E-03
BIOCARTA_IGF1_PATHWAY	0.38	8	21	2571	5.41E-03
BIOCARTA_TFF_PATHWAY	0.38	8	21	2571	5.41E-03
BIOCARTA_HER2_PATHWAY	0.36	8	22	2571	7.48E-03
BIOCARTA_ERK5_PATHWAY	0.39	7	18	2571	8.07E-03

Supplementary Table S8

Pathway	Percentage	num overlap	num in pathway	num miRNA targets	p value
BIOCARTA_CREB_PATHWAY	0.33	9	27	1589	2.95E-04
BIOCARTA_BIOPEPTIDES_PATHWAY	0.24	10	42	1589	2.56E-03
BIOCARTA_EIF4_PATHWAY	0.29	7	24	1589	3.32E-03
BIOCARTA_CACAM_PATHWAY	0.38	5	13	1589	3.43E-03
BIOCARTA_CBL_PATHWAY	0.38	5	13	1589	3.43E-03
BIOCARTA_ARENRF2_PATHWAY	0.38	5	13	1589	3.43E-03
BIOCARTA_MAPK_PATHWAY	0.17	15	87	1589	7.34E-03
BIOCARTA_PYK2_PATHWAY	0.25	7	28	1589	8.37E-03

Supplementary Table S9

Pathway	Percentage	num overlap	num in pathway	num miRNA targets	p value
synaptic transmission	0.23	87	373	2571	7.05E-07
insulin receptor signaling pathway	0.3	41	136	2571	8.66E-07
regulation of transcription, DNA-dependent	0.17	351	2011	2571	2.30E-06
axon guidance	0.24	74	312	2571	2.44E-06
in utero embryonic development	0.26	51	193	2571	3.58E-06
post-embryonic development	0.35	26	74	2571	3.93E-06
regulation of small GTPase mediated signal transduction	0.27	46	171	2571	6.32E-06
transcription, DNA-dependent	0.2	135	688	2571	2.09E-05
nerve growth factor receptor signaling pathway	0.24	53	217	2571	2.66E-05
signal transduction	0.18	235	1324	2571	4.25E-05
small GTPase mediated signal transduction	0.22	72	326	2571	4.36E-05
positive regulation of transcription, DNA-dependent	0.2	105	518	2571	4.46E-05
positive regulation of glucose import	0.48	12	25	2571	4.84E-05
protein phosphorylation	0.2	113	570	2571	6.22E-05
learning	0.41	14	34	2571	9.40E-05
neuron projection development	0.32	21	66	2571	1.68E-04
positive regulation of transcription from RNA polymerase II promoter	0.19	106	545	2571	2.21E-04
neurotransmitter transport	0.38	14	37	2571	2.73E-04
positive regulation of osteoblast differentiation	0.37	14	38	2571	3.78E-04
heart development	0.24	41	173	2571	4.05E-04
intracellular signal transduction	0.21	66	316	2571	4.72E-04
chloride transport	0.3	20	66	2571	4.89E-04
stem cell maintenance	0.47	9	19	2571	4.96E-04
learning or memory	0.36	14	39	2571	5.14E-04
negative regulation of cell migration	0.32	18	57	2571	5.32E-04
protein dephosphorylation	0.25	32	127	2571	5.48E-04
regulation of long-term neuronal synaptic plasticity	0.43	10	23	2571	5.66E-04
skeletal system morphogenesis	0.36	13	36	2571	7.58E-04
nervous system development	0.2	79	402	2571	9.71E-04
palate development	0.31	16	51	2571	1.15E-03
positive regulation of phosphatidylinositol 3-kinase cascade	0.4	10	25	2571	1.24E-03
epidermal growth factor receptor signaling pathway	0.28	21	76	2571	1.36E-03
sodium ion transport	0.25	29	118	2571	1.48E-03
ion transport	0.18	104	563	2571	1.60E-03
fibroblast growth factor receptor signaling pathway	0.27	20	73	2571	1.96E-03
positive regulation of protein phosphorylation	0.26	22	84	2571	2.25E-03
phospholipid biosynthetic process	0.3	15	50	2571	2.67E-03

lung development	0.25	24	96	2571	2.85E-03
collagen fibril organization	0.34	11	32	2571	2.99E-03
BMP signaling pathway	0.29	15	51	2571	3.30E-03
neurotransmitter secretion	0.29	15	51	2571	3.30E-03
blood vessel development	0.29	16	56	2571	3.38E-03
induction of apoptosis by extracellular signals	0.24	25	103	2571	3.61E-03
central nervous system development	0.24	27	114	2571	3.69E-03
spinal cord development	0.4	8	20	2571	3.81E-03
protein ubiquitination	0.22	38	176	2571	3.83E-03
activation of JUN kinase activity	0.33	11	33	2571	3.93E-03
negative regulation of transcription from RNA polymerase II promoter	0.19	71	375	2571	4.46E-03
calcium ion transmembrane transport	0.3	13	43	2571	4.66E-03
transforming growth factor beta receptor signaling pathway	0.26	18	68	2571	4.80E-03
filopodium assembly	0.36	9	25	2571	4.99E-03
positive regulation of Rho GTPase activity	0.36	9	25	2571	4.99E-03
negative regulation of transcription, DNA-dependent	0.19	77	414	2571	5.05E-03
positive regulation of Wnt receptor signaling pathway	0.41	7	17	2571	5.60E-03
calcium ion transport	0.24	24	101	2571	5.71E-03
visual learning	0.33	10	30	2571	5.88E-03
positive regulation of neuron projection development	0.31	11	35	2571	6.50E-03
positive regulation of bone mineralization	0.35	9	26	2571	6.70E-03
lung alveolus development	0.32	10	31	2571	7.61E-03
response to interleukin-1	0.32	10	31	2571	7.61E-03
response to insulin stimulus	0.26	17	66	2571	8.07E-03
regulation of G-protein coupled receptor protein signaling pathway	0.31	11	36	2571	8.20E-03
interspecies interaction between organisms	0.19	59	312	2571	9.14E-03
protein autophosphorylation	0.23	22	94	2571	9.50E-03
regulation of calcium ion transport via voltage-gated calcium channel ac	0.31	10	32	2571	9.70E-03
actin cytoskeleton organization	0.22	28	128	2571	9.91E-03

Supplementary Table S10

Pathway	Percentage	num overlap	num in pathway	num miRNA targets	p value
regulation of transcription, DNA-dependent	0.12	237	2011	1589	2.21E-07
synaptic transmission	0.16	60	373	1589	1.95E-06
transcription, DNA-dependent	0.13	92	688	1589	1.62E-05
heart development	0.18	32	173	1589	3.08E-05
positive regulation of transcription from RNA polymerase II promoter	0.13	73	545	1589	1.13E-04
nervous system development	0.14	57	402	1589	1.40E-04
post-embryonic development	0.23	17	74	1589	1.53E-04
nerve growth factor receptor signaling pathway	0.16	35	217	1589	2.40E-04
in utero embryonic development	0.17	32	193	1589	2.62E-04
potassium ion transport	0.18	27	153	1589	2.78E-04
regulation of long-term neuronal synaptic plasticity	0.35	8	23	1589	4.62E-04
axon guidance	0.14	45	312	1589	4.66E-04
cell differentiation	0.13	68	527	1589	5.53E-04
cellular calcium ion homeostasis	0.22	15	68	1589	5.83E-04
gastrulation with mouth forming second	0.37	7	19	1589	7.09E-04
skeletal system morphogenesis	0.28	10	36	1589	7.14E-04
calcium ion transmembrane transport	0.26	11	43	1589	8.40E-04
calcium ion transport	0.19	19	101	1589	9.61E-04
neuron projection development	0.21	14	66	1589	1.32E-03
insulin receptor signaling pathway	0.17	23	136	1589	1.38E-03
multicellular organismal development	0.11	104	908	1589	1.78E-03
intracellular protein transport	0.14	41	300	1589	2.30E-03
signal transduction	0.11	143	1324	1589	2.86E-03
ion transport	0.12	68	563	1589	3.05E-03
visual learning	0.27	8	30	1589	3.20E-03
cellular membrane fusion	0.29	7	24	1589	3.32E-03
intracellular signal transduction	0.13	42	316	1589	3.44E-03
regulation of transcription from RNA polymerase II promoter by nuclear hormone receptor	0.21	12	58	1589	3.54E-03
neurotransmitter secretion	0.22	11	51	1589	3.67E-03
palate development	0.22	11	51	1589	3.67E-03
chloride transport	0.2	13	66	1589	3.85E-03
central nervous system development	0.17	19	114	1589	4.08E-03
negative regulation of transcription, DNA-dependent	0.13	52	414	1589	4.09E-03
steroid hormone mediated signaling pathway	0.21	11	52	1589	4.29E-03
regulation of gene expression	0.19	13	67	1589	4.40E-03
interspecies interaction between organisms	0.13	41	312	1589	4.66E-03
protein ubiquitination	0.15	26	176	1589	4.91E-03
negative regulation of transforming growth factor beta receptor signaling pathway	0.25	8	32	1589	4.92E-03
positive regulation of mitotic cell cycle	0.3	6	20	1589	5.56E-03
sodium ion transport	0.16	19	118	1589	5.98E-03
lactation	0.24	8	33	1589	6.01E-03
protein phosphorylation	0.12	67	570	1589	6.12E-03

amino acid transport	0.23	9	40	1589	6.18E-03
memory	0.23	9	40	1589	6.18E-03
negative regulation of transcription from RNA polymerase II promoter	0.13	47	375	1589	6.37E-03
regulation of membrane potential	0.24	8	34	1589	7.28E-03
muscle organ development	0.17	16	96	1589	7.99E-03
mammary gland development	0.25	7	28	1589	8.37E-03
negative regulation of translation	0.25	7	28	1589	8.37E-03
fibroblast growth factor receptor signaling pathway	0.18	13	73	1589	9.25E-03
negative regulation of interleukin-6 production	0.31	5	16	1589	9.35E-03
protein methylation	0.31	5	16	1589	9.35E-03
